# Cumulative acquisition of pathogenicity islands has shaped virulence potential and contributed to the emergence of LEE-negative Shiga toxin-producing *Escherichia coli* strains

**DOI:** 10.1080/22221751.2019.1595985

**Published:** 2019-03-29

**Authors:** David Arturo Montero, Felipe Del Canto, Juliana Velasco, Rocío Colello, Nora Lia Padola, Juan Carlos Salazar, Carla San Martin, Angel Oñate, Jorge Blanco, David A. Rasko, Carmen Contreras, Jose Luis Puente, Flemming Scheutz, Eelco Franz, Roberto M. Vidal

**Affiliations:** a Programa de Microbiología y Micología, Instituto de Ciencias Biomédicas, Facultad de Medicina, Universidad de Chile, Santiago, Chile; b Servicio de Urgencia Infantil, Hospital Clínico de la Universidad de Chile “Dr. José Joaquín Aguirre”, Santiago, Chile; c Centro de Investigación Veterinaria Tandil, CONICET-CIC, Facultad de Ciencias Veterinarias, UNCPBA, Tandil, Argentina; d Departamento de Microbiología, Facultad de Ciencias Biológicas, Universidad de Concepción, Concepción, Chile; e Laboratorio de Referencia de E. coli, Facultad de Veterinaria, Universidad de Santiago de Compostela, Lugo, España; f Department of Microbiology and Immunology, University of Maryland School of Medicine, Baltimore, MD, USA; g Departamento de Microbiología Molecular, Instituto de Biotecnología, Universidad Nacional Autónoma de México, Cuernavaca, México; h Department of Bacteria, Parasites and Fungi, The International Collaborating Centre for Reference and Research on Escherichia and Klebsiella, Statens Serum Institut, Copenhagen, Denmark; i National Institute for Public Health, Centre for Infectious Disease Control, Bilthoven, The Netherlands; j Instituto Milenio de Inmunología e Inmunoterapia, Facultad de Medicina, Universidad de Chile, Santiago, Chile

**Keywords:** LEE-negative STEC, Pathogenicity Island, comparative genomics, Integrative Conjugative Element, Locus of Adhesion and Autoaggregation

## Abstract

Shiga toxin-producing *Escherichia coli* (STEC) are foodborne pathogens causing severe gastroenteritis, which may lead to hemolytic uremic syndrome. The Locus of Enterocyte Effacement (LEE), a Pathogenicity Island (PAI), is a major determinant of intestinal epithelium attachment of a group of STEC strains; however, the virulence repertoire of STEC strains lacking LEE, has not been fully characterized. The incidence of LEE-negative STEC strains has increased in several countries, highlighting the relevance of their study. In order to gain insights into the basis for the emergence of LEE-negative STEC strains, we performed a large-scale genomic analysis of 367 strains isolated worldwide from humans, animals, food and the environment. We identified uncharacterized genomic islands, including two PAIs and one Integrative Conjugative Element. Additionally, the Locus of Adhesion and Autoaggregation (LAA) was the most prevalent PAI among LEE-negative strains and we found that it contributes to colonization of the mice intestine. Our comprehensive and rigorous comparative genomic and phylogenetic analyses suggest that the accumulative acquisition of PAIs has played an important, but currently unappreciated role, in the evolution of virulence in these strains. This study provides new knowledge on the pathogenicity of LEE-negative STEC strains and identifies molecular markers for their epidemiological surveillance.

## Introduction

Shiga toxin-producing *Escherichia coli* (STEC) are a group of food-borne pathogens that cause diarrhea and severe diseases, such as Hemorrhagic Colitis (HC) and Hemolytic Uremic Syndrome (HUS), primarily in children under 5 years of age [1]. STEC causes outbreaks and sporadic cases of gastroenteritis affecting both public health and the food industry [2]. Shiga toxins (Stx) are the primary STEC virulence factors responsible for pathologies such as HC and HUS [3]. There are two variants of Stx, Stx1 and Stx2, which include subtypes (Stx1a,c,d and Stx2a-g) defined by nucleotide differences, biological activity and serological reactivity [4]. STEC strains producing Stx1a, Stx2a, and/or Stx2d are associated with the most severe cases [5].

The Locus of Enterocyte Effacement (LEE) Pathogenicity Island (PAI) harbors genes that mediate the adhesion phenotype of a group of STEC strains (LEE-positive) that are associated with HUS and are clinically relevant. Specifically, LEE mediated adherence leads to the loss of intestinal microvilli, resulting in severe diarrhea [6]. However, STEC strains lacking LEE (LEE-negative STEC) have also been isolated from severe disease cases, suggesting the existence of additional virulence factors that also favor the pathogenicity of these bacteria. A study examining a small number of STEC LEE-negative strains demonstrated that this is a heterogeneous group, harboring diverse arrays of virulence factors [7]. Genetic elements such as the PAIs harbor several of these virulence factors. Three PAIs have been reported as exclusively present in LEE-negative STEC strains, namely: the Locus of Proteolysis Activity (LPA) [8], the Subtilase-Encoding Pathogenicity Island (SE-PAI) [9] and the Locus of Adhesion and Autoaggregation (LAA) [10]. However, the pathogenicity mechanisms mediated by these PAIs have not been thoroughly investigated. Recently, LEE-negative STEC strains belonging to serogroups O91, O113, O128, O146 and O174 have been increasingly identified from clinical cases in Europe, Argentina and South Korea [11–15]. Unfortunately, limited surveillance of LEE-negative STEC strains has prevented an accurate assessment of their global spread and impact on public health [16].

A useful strategy for studying the evolution of these pathogens involves combining comparative genomics with epidemiological data [17,18]. Moreover, associations between genetic data and phenotypic behavior improve these analyses and may contribute to reaching new insights into the features that have favored the emergence of these strains in the clinical setting, as well as delineate the virulence potential of specific strains [19]. While studies using these approaches have increased our understanding of LEE-positive STEC strains [17,19,20], the number of studies involving LEE-negative STEC strains is limited [7].

Therefore, we performed a large-scale genomic analysis of 367 LEE-negative STEC strains isolated globally from several sources, including humans, food, animals and the environment. As a result, we were able to identify uncharacterized Genomic Islands (GIs), including two novel PAIs and one Integrative and Conjugative Element (ICE). In addition, we found that LAA was the most prevalent PAI among LEE-negative STEC strains, suggesting that it plays an important role. This work also provides experimental evidence supporting the participation of LAA in the intestinal colonization of a mouse model of STEC infection. Phylogenetic analyses and genomic comparisons suggest that evolutionary events, in which individual genes or groups of genes are acquired, could explain the increased incidence of these strains. This study is a step forward in our knowledge of the evolution of genomes and pathogenic mechanisms of LEE-negative STEC, with important implications for future studies on their epidemiology and surveillance.

## Materials and methods

### Bacterial strains, plasmids and primers

The STEC strains used in this study are summarized in Table S1. Spontaneously derived streptomycin resistant (Str^r^) mutants of STEC strains O113:H21 E045-00 and O91:H21 V07-4-4, were used in this study. Strains were grown in static or agitated LB at 37°C. Culture media was supplemented as needed with ampicillin (100 µg/ml), kanamycin (50 µg/ml) or streptomycin (50 µg/ml). Plasmids and primers used are summarized in Table S9.

### Genome sequencing

The 35 STEC strains sequenced in this study were isolated from Argentina (*n* = 14) [21], Chile (*n* = 16) [22] and Spain (*n* = 5). Genomic DNA was extracted using the Wizard genomic DNA purification kit (Promega Corp., USA) and sequenced at MicrobesNG (University of Birmingham, UK) using Illumina MiSeq or HiSeq 2500 technology platforms with 2×250-bp paired-end reads. Draft genomes were provided after trimming low quality-ends and assembling reads with SPAdes 3.10 [23]. Assembly statistics were obtained with Quast v4.6.3 [24]. Contigs shorter that 200 nt were removed and sequences were deposited at GenBank under Bioproject PRJNA448751. Nucleotide accession codes are included in Table S1.

### Publicly available genome sequences

A total of 332 genome sequences of LEE-negative STEC strains were downloaded from GenBank on 15 January 2018. Sequences management and BLAST searches were performed using Geneious software (v11.0.5; Biomatters Ltd). The presence of *stx* genes and the absence of the *eae* gene were confirmed using BLASTn. Genomes accession numbers are listed in Table S1.

### *In silico* serotyping and detection of virulence genes

Serotype was determined *in silico* using the SerotypeFinder tool [25]. A set of virulence genes (*stx, subA, astA, cdtB, sta1, stb, senB, lpfA, iha, espP, espI, epeA, pic, sigA, cba, celb, cma, mcmA, mchB, mchC and mchF*) were identified using VirulenceFinder [26]. Other genes were detected using BLASTn: *saa* (AF399919.3; positions:6290-7840), *sab* (AY258503.2; positions:118905-123200), *eibG* (GU295813.1), *hes* (CP023541.1; positions:4035512-4036252), *lesP* (CP023541.1; positions:4063646-4067740), *tia* (JQ994271.1; positions:4726-5472), and *hra1* (U07174.1). The presence of a gene was confirmed when a coverage and identity >90% was found. The *ag43* gene has several alleles that are divided into two subfamilies [27]. Therefore, the identification of the *ag43-I* (subfamily I) and *ag43-II (*subfamily II) alleles was achieved by *in silico* PCR using primers targeting conserved regions as previously described by Montero et al. [22].

### Identification of PAIs and ICEs

Nucleotide sequences of LAA, SE-PAI, LPA and the High-Pathogenicity Island (HPI) were downloaded from GenBank, using accession numbers AFDQ01000026.1 (positions:385984-472336), JQ994271, AJ278144.1 and AL031866 (positions: 78113-114560), respectively. These sequences were used to perform BLASTn searches against each genome. By default, absence was defined as coverage and/or identity <70%. Since LAA is composed of four modules [10], a strain was considered positive for this PAI when at least three of its modules were identified. Identification and characterization of genomic regions with features of PAIs and/or ICEs were performed using REPuter [28], ISfinder [29] and tRNAscan-SE [30]. Open reading frames (ORFs) and the G+C content were determined using Geneious software. Contigs assembly (mapping) against a reference sequence was performed in Geneious software. The genomic regions corresponding to new PAIs and ICEs were extracted from the original genome sequences available in GenBank and then annotated using the RAST server [31] (Files S1-3). These genomic regions were searched in the Islander [32] and PAIDB [33] databases but none of the homologous sequences exceeded 20% of consultation coverage. The association network (based on a presence/absence matrix) showing the co-occurrence or mutually exclusive patterns in the distribution of PAIs was drawn using igraph package [34] in R [35]. For the presence/absence matrix, absence was defined as coverage and/or identity <70%. Comparisons between ICE sequences were performed using progressiveMauve [36].

### Construction of isogenic mutants

The LAA PAI was inactivated in the STEC E045-00 and V07-4-4 genomes by allelic replacement, as previously described [37]. Both strains have the complete set of LAA’s genes compared to the prototype LAA_B2F1_ and the deletion comprised approximately 86 kb. For this, the E045-00 and V07-4-4 strains carrying plasmid pKD46, encoding the lambda-derived Red recombination system, were transformed with a PCR fragment containing the kanamycin resistance (Kan) gene flanked by 49 and 53 nucleotides identical to the 3′-ends of the *pheV-tRNA* and the LAA PAI (Table S9). The kanamycin-resistant clones were analyzed by PCR to verify the allelic exchange at the right locus using an internal primer to the Kan gene (Kan_rev) and another flanking LAA (LAA_conf_for).

### Two-dimensional polyacrylamide gel electrophoresis

Outer membrane protein (OMP) extracts were obtained and analyzed by 2D-PAGE as described [22]. Selected spot proteins were cut from the polyacrylamide gels and identified by MALDI-TOF/TOF at the Mass Spectrometry Core at the University of Texas Medical Branch (Galveston, Texas).

### Bacterial adhesion and biofilm formation assays

Bacterial adhesion to Caco-2 and HT-29 epithelial cells and biofilm formation were measured as previously described [10].

### Mouse infection studies

All animal experiments were carried out at the Universidad de Concepción, following protocols approved by the Faculty of Biological Sciences Bioethics Committee. Male BALB/c mice (6–8 weeks old; from Instituto de Salud Pública, Santiago, Chile) were randomly distributed into experimental groups. Mice were kept in conventional animal facilities and received water and food *ad libitum*. The STEC infection mouse model was carried out as previously described [38], with minor modifications. Briefly, mice were given water *ad libitum* containing streptomycin (5 g/l) 24–48 h prior to inoculation and for the duration of the experiment. Bacterial strains were grown in agitated LB supplemented with streptomycin (50 µg/ml) at 37°C for 18 h. Cultures were centrifuged, the pellets were washed in sterile PBS and resuspended in a 20% sucrose (w/v) and 10% NaHCO_3_ (w/v) solution in sterile water to ∼10^10^ colony forming units (cfu)/ml. Bacterial concentration was confirmed by serial dilution and cfu counts in LB agar plates.

Mice were then starved of food and water overnight (12 h). The next morning, each animal was orally infected by pipette feeding with 100 µl of a bacterial suspension containing 10^9^ cfu/ml. Following challenge, animals were housed individually, and food and water supplemented with streptomycin (5 g/l) was reintroduced and provided *ad libitum*. Intestinal colonization by the challenging bacteria was determined by bacterial shedding in feces over time. Feces were collected daily, weighed, homogenized, suspended in 1 ml PBS and, after serial dilutions in PBS, plated on MacConkey agar plates supplemented with streptomycin (50 µg/ml) to determine the cfu. For the survival experiments mice were handled and infected as described for the colonization experiments’ however, the cfu in feces over time was not recorded.

### Phylogeny, phylogroups, molecular typing and Bayesian analysis of population structure (BAPS)

A parsimony phylogenetic tree based on core single nucleotide polymorphisms (SNPs) of 367 complete or draft genomic sequences of LEE-negative STEC, and *E. coli* K-12 MG1655, was built with kSNP3.1 [39], using a 20-core Dell Poweredge R730 server. Briefly, SNPs flanked by stretches of six nucleotides on each side were identified using k-mers of 13-nucleotides, screened in all the genomes, counted and tabulated into a matrix, in order to build a phylogenetic tree with the parsimony method. A total of 3,956 SNPs were considered for the final tree, which represents the consensus of 100 bootstrap replicates. The tree was further processed with the Interactive Tree of Life tool [40]. LEE-negative STEC population structure was delineated with RhierBAPS [41] using the 3,956 core SNPs. For this, a 3 depth levels and a maximum clustering size of 70 (default = number of isolates/5; 367/5 = 73,4) were specified. Phylogroup assignment was conducted *in silico* using the ClermonTyping Tool [42]. STs of each strain were determined according to the Achtman scheme [43] using MLST 1.8 tool [44].

### Statistical analysis

Differences in the distribution of virulence genes between isolation sources were analyzed using Fisher's Exact test (two-tailed). Pairwise association (co-occurrence or mutual exclusivity) between PAIs, ICEs and toxins was performed in contingency tables by Odds ratios. The statistical significance of these associations was determined using Pearson's chi-squared test or Fisher's exact test (when frequencies were less than 5). When any of the cell values of the contingency table was zero, 0.5 was added to all cells (Haldane correction) to avoid errors in the statistical test. Differences in bacterial colonization and survival rates between groups of mice were analyzed using Mann–Whitney *U* Test and log-rank test, respectively. Differences between the genome sizes were analyzed using Kruskal–Wallis test followed by Dunn's multiple comparison test. For all statistical tests, a *P* value of <0.05 was considered significant.

## Results

### Strains analyzed in this study

Genomes of thirty-five LEE-negative STEC strains isolated in Argentina (*n* = 14), Chile (*n* = 16) and Spain (*n* = 5) were sequenced and analyzed along genomes (draft or complete) of 332 LEE-negative STEC strains isolated worldwide that are available in GenBank (Table S1, Figure S1a). These strains were isolated from humans (*n* = 150), animals (*n* = 123), food (*n* = 45), the environment (*n* = 4), and from unknown sources (*n* = 45) (Table S2) between 1947 and 2016 (Figure S1b).

### Serotypes, toxins and virulence gene content

A total of 101 serotypes were identified using SerotypeFinder tool [25] (Table S1 and S2); however, in 15 genomes only the ‘H’ antigen was identified. The most frequent serotypes were O91:H14 (*n* = 29), O113:H21 (*n* = 29), O91:H21 (*n* = 22), O174:H21 (*n* = 18), O146:H21 (*n* = 17), O174:H8 (*n* = 12), O22:H8 (*n* = 10) and O8:H19 (*n* = 9), which have been previously reported to cause diarrhea, dysentery and HUS [16,45–47]. Previous studies have also demonstrated this significant diversity of O:H types among LEE-negative STEC strains [10,45].

The presence/absence of *stx* genes and other virulence genes harbored by these strains was determined using VirulenceFinder tool [26] and the BLASTn algorithm (Table S1 and S3). The frequency of detection for each gene is presented in Table 1. Among the strains (*n* = 367), 266 (72.5%) contained only one *stx* gene and 101 (27.5%) had more than one (Table S4). The most frequent combinations of *stx* genes were *stx_1a_* + *stx_2a_* (32/367; 8.7%) and *stx_1c_* + *stx_2b_* (30/367; 8.2%). The *stx_2b_* gene was found in a statistically greater proportion of human strains compared to animal strains. In contrast, *stx_1d_*, *stx_2a_* and *stx_2e_* were found in a greater proportion of animal strains compared to human strains. Additionally, the virulence genes *senB, eibG, ag43-I, tia and sigA* were significantly more frequent in human strains, whereas *sta1*, *saa, hra1, lpfA, lesP and espP* were significantly more often found in animal strains.

**Table 1.** Distribution of virulence determinants among LEE-negative STEC strains isolated from different sources.

Gene	Total frequency (*n* = 367), *n* (%)	Source of isolation, *n* (%) ^a^				*p* value ^b^	
		Human (*n* = 150)	Animal (*n* = 123)	Food (*n* = 45)	Unidentified (*n* = 45)	Human vs. Animal	Human vs. Food
Shiga toxin subtypes^c^							
*stx_1a_*	101 (27.5)	50 (33.3)	35 (28.5)	8 (17.8)	6 (13.3)	0.431	0.060
*stx_1c_*	71 (19.3)	36 (24.0)	20 (16.3)	8 (17.8)	7 (15.6)	0.133	0.424
*stx_1d_*	10 (2.7)	1 (0.7)	**6(4.9)**	0 (0.0)	3 (6.7)	**0.048**	–
*stx_2a_*	105 (28.6)	27 (18.0)	**44(35.8)**	**19(42.2)**	12 (26.7)	**0.001**	**0.001**
*stx_2b_*	62 (16.9)	**35(23.3)**	15 (12.2)	3 (6.7)	9 (20.0)	**0.019**	**0.017**
*stx_2c_*	33 (9.0)	17 (11.3)	8 (6.5)	4 (8.9)	4 (8.9)	0.208	0.788
*stx_2d_*	56 (15.3)	25 (16.7)	17 (13.8)	5 (11.1)	9 (20.0)	0.614	0.482
*stx_2e_*	16 (4.4)	2 (1.3)	**10(8.1)**	1 (2.2)	3 (6.7)	**0.007**	0.081
*stx_2g_*	7 (1.9)	0 (0.0)	3 (2.4)	8 (2.2)	3 (6.7)	–	–
Other toxin genes							
*ehxA*	179 (48.8)	75 (50.0)	60 (48.8)	25 (55.6)	15 (33.3)	0.903	0.611
*subA*	130 (35.4)	60 (40.0)	40 (32.5)	14 (31.1)	14 (31.1)	0.209	0.299
*cdtB*	53 (14.4)	15 (10.0)	22 (17.9)	9 (20.0)	7 (15.6)	0.075	0.117
*astA*	62 (16.9)	20 (13.3)	21 (17.1)	9 (20.0)	12 (26.7)	0.400	0.338
*sta1*	23 (6.3)	3 (2.0)	**11(8.9)**	**7(15.6)**	1 (2.2)	**0.012**	**0.001**
*stb*	4 (1.1)	1 (0.7)	2 (1.6)	0 (0.0)	1 (2.2)	0.590	–
*senB*	35 (9.5)	**26(17.3)**	5 (4.1)	1 (2.2)	2 (4.4)	**0.001**	**0.012**
Molecular markers of LEE-negative STEC^d^							
*saa*	108 (29.4)	33 (22.0)	**50(40.7)**	13 (28.9)	9 (20.0)	**0.001**	0.423
*hes*	150 (40.9)	61 (40.7)	57 (46.3)	16 (35.6)	15 (33.3)	0.390	0.604
*eibG*	33 (9.0)	**27(18.0)**	1 (0.8)	1 (2.2)	4 (8.9)	**<0.001**	**0.013**
*sab*	17 (4.6)	9 (6.0)	6 (4.9)	2 (4.4)	0 (0.0)	0.793	1.000
Adhesins							
*tia*	121 (33)	**70(46.7)**	27 (22.0)	11 (24.4)	12 (26.7)	**<0.001**	**0.009**
*hra1*	43 (11.7)	8 (5.3)	**20(16.3)**	6 (13.3)	9 (20.0)	**0.004**	0.095
*lpfA*	309 (84.2)	120 (80.0)	**111(90.2)**	37 (82.2)	37 (82.2)	**0.027**	0.832
*Iha*	273 (74.4)	126 (84.0)	91 (74.0)	29 (64.4)	24 (53.3)	0.0502	**0.006**
*ag43-I*	277 (75.5)	**128(85.3)**	88 (71.5)	24 (53.3)	33 (73.3)	**0.007**	**<0.001**
*ag43-II*	96 (26.2)	36 (24.0)	29 (23.6)	16 (35.6)	14 (31.1)	1.000	0.177
Serine Protease Autotransporters of Enterobacteriaceae (SPATEs)							
*lesP*	140 (38.1)	48 (32.0)	**57(46.3)**	17 (37.8)	17 (37.8)	**0.018**	0.589
*espP*	94 (25.6)	28 (18.7)	**40(32.5)**	**15(33.3)**	8 (17.8)	**0.011**	**0.043**
*espI*	61 (16.6)	31 (20.7)	19 (15.4)	5 (11.1)	6 (13.3)	0.340	0.219
*epeA*	16 (4.4)	10 (6.7)	4 (3.3)	1 (2.2)	1 (2.2)	0.319	0.311
*pic*	14 (3.8)	8 (5.3)	2 (1.6)	1 (2.2)	2 (4.4)	0.120	0.469
*sigA*^e^	17 (4.6)	**16(10.7)**	0 (0.0)	0 (0.0)	1 (2.2)	–	–
Colicins							
*cma*	52 (14.2)	22 (14.7)	12 (9.8)	9 (20.0)	9 (20.0)	0.270	0.485
*cba*	70 (19.1)	29 (19.3)	22 (17.9)	9 (20.0)	10 (22.2)	0.876	1.000
*celb*	93 (25.3)	31 (20.7)	36 (29.3)	10 (22.2)	13 (28.9)	0.120	0.836
Microcins							
*mcmA*	16 (4.4)	9 (6.0)	5 (4.1)	0 (0.0)	2 (4.4)	0.585	–
*mchB*	76 (20.7)	**57(38.0)**	10 (8.1)	2 (4.4)	7 (15.6)	**<0.001**	**<0.001**
*mchC*	75 (20.4)	**56(37.3)**	10 (8.1)	2 (4.4)	7 (15.6)	**<0.001**	**<0.001**
*mchF*	90 (24.5)	**60(40.0**)	17 (13.8)	5 (11.1)	8 (17.8)	**<0.001**	**<0.001**
PAIs and ICEs							
LAA	151 (41.2)	60 (40)	57 (46.3)	16 (35.6)	17 (37.8)	0.326	0.607
SE-PAI	65 (17.7)	**38(25.3)**	14 (11.4)	4 (8.9)	9 (20)	**0.005**	**0.022**
LPA	59 (16.1)	31 (20.7)	18 (14.6)	4 (8.9)	6 (13.3)	0.208	0.079
HPI	43 (11.7)	14 (16)	10 (8.1)	5 (11.1)	3 (6.7)	0.831	0.775
LIC	25 (6.8)	15 (10)	4 (3.3)	6 (13.3)	0 (0.0)	**0.032**	0.580
LAC	11 (2.9)	6 (4.0)	3 (2.4)	2 (4.4)	0 (0.0)	0.520	1.000
ICE*Ec*8	12 (3.2)	**12(8.0)**	0 (0.0)	0 (0.0)	0 (0.0)	–	–
Undetermined	106 (28.9)	28 (18.7)	**38(30.9)**	**19(42.2)**	19 (42.2)	**0.023**	**0.001**

^a^Strains isolated from the environmental are not shown due to their small number (*n* = 4).

^b^*P* values were obtained by Fisher's Exact test (two-tailed) comparing strains isolated from humans, animals or food. A *p* value <0.05 was considered significant. Significant values are shown in bold.

^c^None of the strains was positive for the *stx_2f_* gene.

^d^These genes are exclusively present in LEE negative STEC strains and therefore they are considered as molecular markers of this group of bacteria.

^e^The *sigA* gene was almost exclusively present in strains isolated from humans.

A previous study reported the presence of genes encoding bacteriocins among LEE-negative STEC strains isolated from humans [11]. Bacteriocins are antimicrobial peptides produced by bacteria that are active against closely related strains [48]. *E. coli* produces two types of bacteriocins, classified by their molecular weight as colicins (25–80 kDa) and microcins (<10 kDa) [48]. Notably, we found that the *mch* cluster involved in the synthesis of microcins was more frequently detected in strains isolated from humans compared to strains isolated from other sources (Table 1). Whether the production of microcins improves the fitness of these bacteria during human infection remains to be investigated. Altogether, these data show that LEE-negative STEC strains contain several genes that potentially provide phenotypes associated with adhesion, colonization and toxicity. While different combinations of some of these genes may contribute to virulence during human infection, others may be necessary for persistence in animal reservoirs.

### Identification of novel Pathogenicity Islands and Integrative and Conjugative Elements

**Figure 1.**
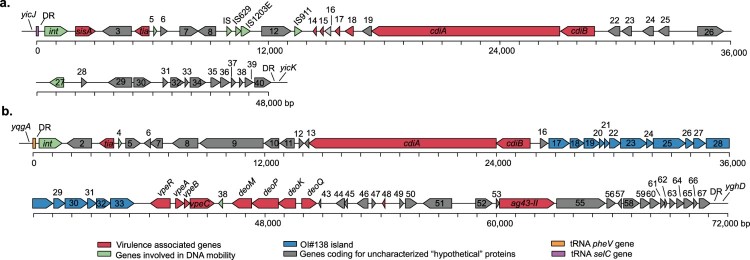
Genetic structure of two new Pathogenicity Islands identified among LEE-negative STEC strains. Predicted genes and direction of transcription are represented as block arrows. Open reading frames (ORFs) are color coded according to gene function (see legend). Names of some genes are shown. Features of each ORF are listed in Tables S5 and S6. (a) Locus of Invasion and Contact-dependent Growth Inhibition (LIC). This genomic region is located adjacent to the *selC*-tRNA gene in the contig 13 (GenBank accession: NJVC01000013) of the draft genome of STEC O174:H21 str. MOD1-EC1633. The complete LIC sequence is available in File S1 (b) Locus of Adhesion and Colonization (LAC). This genomic region is located adjacent to the *pheV*-tRNA gene in the contig 3 (GenBank accession: AYKD01000003.1) of the draft genome of *E. coli* strain FCH1. The complete LAC sequence is available in File S2.

PAIs contribute to the evolution and virulence of pathogenic *E. coli* strains [49]. Therefore, we aimed to determine the distribution of the SE-PAI, LPA and LAA islands. We also searched for the HPI of *Yersinia pestis*, which is widely present among many Enterobacteriaceae, including *E. coli* [50]. Several of the virulence genes highlighted in this study have been previously mapped to known PAIs, such as *tia* and *subA* in SE-PAI; *iha* and *espI* in LPA; and *hes*, *iha*, *lesP* and *ag43*-I in LAA. Interestingly, BLASTn searches using the complete nucleotide sequences of the PAIs showed that *tia* is also present in other regions of the chromosome of strains that lack SE-PAI. Further analysis on the genomic context of *tia* revealed two previously uncharacterized genomic regions which contained this gene and that exhibit features commonly found in PAIs (Figure 1).

One of these PAIs, found in contig 13 of the draft genome of STEC O174:H21 MOD1-EC1633 (GenBank accession: NJVC01000013), is a 48-kb genomic region located next to the *selC*-tRNA gene (Figure 1(a)). Among other characteristics it includes: (i) an integrase-encoding gene (ORF1) localized downstream of the *selC*-tRNA gene; (ii) it is flanked by 23-bp imperfect direct repeats (DR), corresponding to the 3′ end of the *selC*-tRNA gene; (iii) a lower GC content than average for the MOD1-EC1633 chromosome; (iv) several insertion sequences (IS); and (v) various virulence-associated genes, such as *tia* (ORF4), which has been involved in invasion of LEE-negative STEC strains [51], as well as a group of genes (ORFs 14–21) encoding a member of the contact-dependent growth inhibition (CDI) system [52]. Specifically, ORF20 encodes a protein sharing 89.3% similarity with the prototypic CdiA protein (GenBank protein access: AAZ57198.1) of *E. coli* EC93 [53], while ORF21 encodes a CdiB homolog, a β-barrel protein which exports CdiA through the outer membrane. Since this genomic region contained the *tia* gene related to bacterial invasion and encoded a CDI system, it was named Locus of Invasion and Contact-dependent Growth Inhibition (LIC).

The second PAI was identified in contig 57 of STEC O22:H8 MOD1-EC3763 draft genome (GenBank access: NJSE01000057.1); nevertheless, this contig is only 13-kb and not thought to contain the whole PAI sequence. This genomic region was not found in other LEE-negative STEC strains within a single contig, but an additional search in GenBank identified a sequence with 99.9% identity in contig 3 (214-kb) of the draft genome of *E. coli* FCH1 (GenBank accession: AYKD01000003.1). Further analyses revealed that the 13-kb fragment is part of a 71-kb PAI inserted in the *pheV*-tRNA gene of the FCH1 chromosome (Figure 1(b)). The characteristics of each ORF in this region are shown in Table S6. Subsequently, the contigs of the MOD1-EC3763 strain were mapped against the Whole Genome Sequence (WGS) of strain FCH1 (see Methods), allowing us to identify 99.6% of the PAI (70,923 bp with 99.8% identity) within 7 ordered and concatenated contigs of the draft genome of strain MOD1-EC3763 (Figure S2). The presence of the PAI structure was also determined in other STEC strains (see below). Interestingly, the FCH1 strain is a human isolate lacking *stx* genes [54], indicating that this PAI is not restricted to STEC. The characteristics identified in this genomic region include: (i) it is inserted / integrated next to the *pheV*-tRNA gene; (ii) an integrase-encoding gene (ORF1) located downstream of the *pheV*-tRNA gene; (iii) flanking 23-bp imperfect DR corresponding to the 3′ end of the *pheV*-tRNA gene; (iv) IS elements; and (v) several virulence genes, including *tia* (ORF3); ORF14 encoding a protein sharing 88.1% and 86.5% similarity with the prototypic CdiA protein and the CdiA homolog harbored by the LIC PAI, respectively; the *vpe* (ORFs 34–37) and *deoK* (ORFs 39–42) operons, which are also present in GIs of other *E.coli* pathotypes and promote intestinal colonization by pathogenic *E. coli* strains in murine infection models [55,56]; and ORF54, which encodes a protein sharing 87.6% and 79.8% similarity with Cah (GenBank access: AAG55356.1) and Ag43 (GenBank access: AUG16753.1), which participate in adhesion, autoaggregation and biofilm formation [27]. Moreover, we identified a group of genes (ORFs 17–33) sharing 91% identity with the OI#138 island (GenBank access: AE005571) of STEC O157:H7. The OI#138 has not been previously characterized, but its predicted function is the biosynthesis of fatty acids and polyketides [57]. Since this group of genes is related to adhesion and colonization, this genomic region was named Locus of Adhesion and Colonization (LAC).

**Figure 2.**
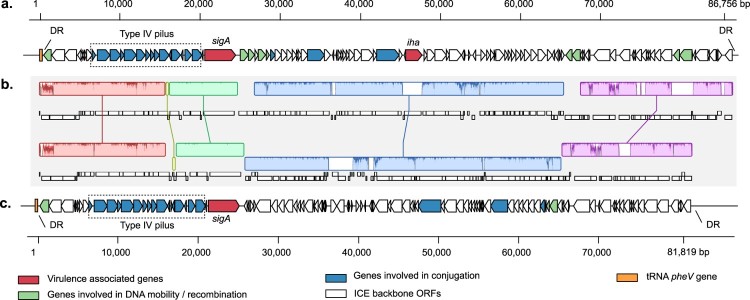
Genetic structure of the Integrative and Conjugative Elements (ICEs) identified among LEE-negative STEC strains. (a) and (c) ICEs identified among LEE-negative STEC strains. (a) ICE located adjacent to *pheV*-tRNA gene in STEC O117:H7 str. FHI72 draft genome (GenBank assembly accession: GCA_000939255.1). The complete ICE*Ec7* sequence is available in File S3. (c) ICE located adjacent to *pheV*-tRNA gene in the contig 26 (GenBank accession: LOGV01000031.1) of the STEC O91:H14 str. 2174 draft genome. Predicted genes and transcription direction are represented as block arrows. Open reading frames (ORFs) are color coded according to gene function, as indicated by legend at the bottom. Names of some genes are shown. Features of each ORF in (a) are listed in Table S7. (b) Alignment between ICEs shown in (a) and (c). Alignment was performed using progressiveMauve [36]. Colored segments represent homologous regions. Non-colored areas represent unaligned sequences that may be genome-specific. Inverted regions are identified by boxes below the central line.

The *sigA* gene was almost exclusively present in a group of clinical LEE-negative STEC strains (Table 1, Table S1). This gene was originally reported in the *she* PAI of *Shigella flexneri* 2a [58] and later in two PAIs harbored by the STEC/EAEC O104:H4 strain that caused the 2011 HUS outbreak in Europe [59]. Considering these observations, we sought to determine whether this gene is harbored in a mobile genetic element (MGE). *In silico* analysis of the genomic context of *sigA* in the draft genome of STEC O117:H7 str. FHI72 (GenBank assembly accession: GCA_000939255.1) showed that an integrase-encoding gene and a *pheV-*tRNA are located 20,168 and 20,442 bp upstream of the *sigA* start codon, respectively (Figure 2(a)). In addition, a 52-bp perfect DR corresponding to the 3′ end of the *pheV-*tRNA was also identified 66,066 bp downstream of the *sigA* start codon. These DR sequences flank an 86,482-bp genomic region inserted in the *pheV*-tRNA gene, which has a similar G+C content (51%) as the FHI72 chromosome. A total of 103 ORFs were identified, including genes involved in DNA conjugation and type IV pilus biogenesis (Table S7), features that are exhibited by integrative and conjugative elements (ICEs) [60]. Seven ICEs had been identified in *E. coli* according to the ICEberg database [61]. Consequently, based on the nomenclature proposed by Burrus et al. [62], we named this genomic region ICE*Ec*8 and found that it is present in strains belonging to the serotypes O91:H14 (7/29; 24%) and O117:H7 (5/5; 100%). In addition to *sigA* (ORF23), another virulence gene carried by ICE*Ec8* of O117:H7 strains is *iha* (ORF52), which is absent in ICE*Ec8* harbored by O91:H14 strains that also has an inverted region of about 40-kb (Figure 2(b,c)).

Overall, our results indicated that the most prevalent PAI was LAA (151/367; 41.2%), followed by SE-PAI (65/367; 17.7%), LPA (59/367; 16.1%) and HPI (43/367; 11.7%). The least frequent PAIs were LIC (25/367; 6.8%) and LAC (11/367; 3%) (Table 1). Regarding to the distribution of ICE*Ec*8, it was identified in 12/367 (3.2%) human strains. All these MGEs were identified in strains belonging to various serotypes (Table S8), demonstrating their widespread distribution among LEE-negative STEC strains; however, only SE-PAI and LIC were detected with a significantly (*p* < 0.05) higher frequency in human strains compared to animal strains (Table 1). Besides, ICE*Ec*8 was exclusively identified in human strains.

**Figure 3.**
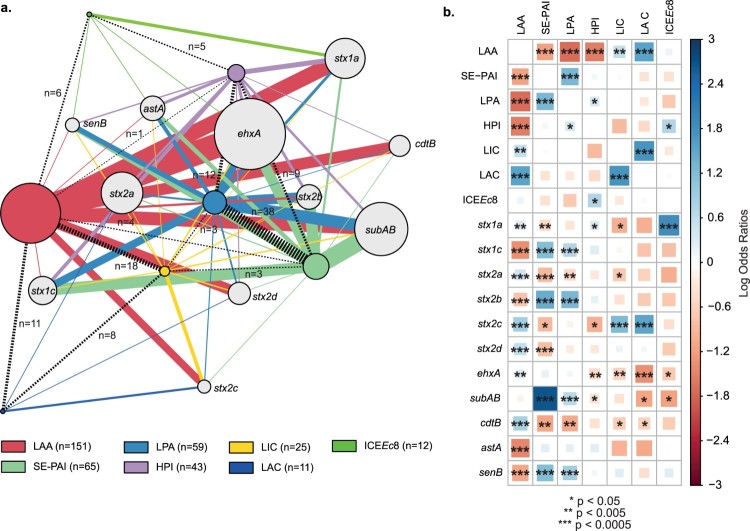
Patterns of association between PAIs, ICEs and toxins identified among the LEE-negative STEC strains (a) Graph of modules showing interactions among PAIs, ICEs and toxins. Module links are weighted by both the number of strains linked between modules and the number of strains within modules. Modules and links of PAIs and ICEs are colored according to the legend. The number of strains positive for each PAI and ICE is shown in parentheses in the legend. The number of strains in which two specific PAIs were identified (co-occurrence) is shown next to the links (dotted lines). The figure was prepared using the package igraph [34] in R [35]. (b) Pairwise association plot for PAIs, ICEs and toxins. Red squares represent negative associations (mutual exclusivity); Blue squares represent positively associations (co-occurrence). The color scale represents the magnitude of the association determined by Pearson's Chi-square test or Fisheŕs exact test (when frequencies were less than 5). The figure was prepared using the package corrplot [76] in R [35].

Additionally, we found patterns of association (co-occurrence and mutual exclusivity) among PAIs, the ICE*Ec*8 and some toxin genes (Figure 3). For instance, LAA, LIC and LAC, SE-PAI and LPA, as well as LPA and HPI, have a high co-occurrence probability (*p* < 0.005, *p* < 0.0005 and *p* < 0.05, respectively) (Figure 3(b)). In contrast, LAA is mutually excluding with SE-PAI, LPA and HPI (*p* < 0.0005). Notably, LAA was co-occurrent with *stx_1a_*, *stx_2a_, stx_2c_* and *stx_2d_*, which encode to the Stx subtypes responsible for the most severe cases of STEC infection [5]. Whether these associations provide selective advantages to particular strains or are linked to the genetic background of each strain is not clear.

### Functional analysis of the LAA PAI

The high prevalence of LAA among LEE-negative STEC strains suggests that it may play an important biological role, but its contribution to the pathogenicity remains uncharacterized. Therefore, we generated a LAA deletion mutant of STEC O113:H21 E045-00 to address its role in different functional *in vitro* and *in vivo* assays.

**Figure 4.**
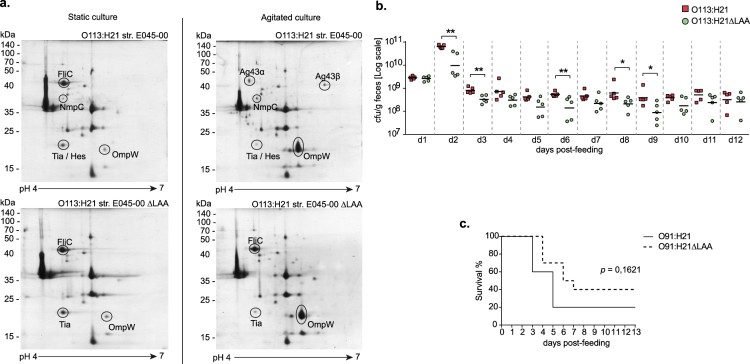
Functional analyses of the LAA PAI. (a) 2D-PAGE OMP profiles of STEC O113:H21 E045-00^SR^ strain and its isogenic mutant ΔLAA^SR^. Strains were grown in LB broth with (right) or without agitation (left). 12% polyacrylamide gels (13 cm; pH range: 4–7) were stained with Coomassie blue G-250, and selected spots (proteins) were identified by MALDI-TOF/TOF mass spectrometry. The scale bars on the left indicate molecular weights in kDa. (b) Colonization of streptomycin-treated mice orally inoculated with 10^9^ cfu of E045-00^SR^ or E045-00ΔLAA^SR^. Fecal pellets were collected daily, weighed, homogenized, and plated on MacConkey agar containing streptomycin. Data points are cfu/g of fecal sample collected from each mouse on the indicated day. Lines represent means. Differences in colonization levels for each day were analyzed using the Mann-Whitney *U* Test (*p < 0.05 **p < 0.005). (c) Survival rate of groups of ten streptomycin-treated mice orally inoculated with STEC O91:H21 V07-4-4^SR^ strain or its isogenic mutant ΔLAA^SR^. The difference in the survival rate between both groups was analyzed using the log-rank test. Statistical significance level was defined as *p* < 0.05.

The OMP profiles of E045-00 and its isogenic mutant ΔLAA, grown in static or agitated LB cultures at 37°C, were analyzed by 2D-PAGE (Figure 4a). Differentially synthesized OMP proteins were identified by mass spectrometry. The LAA-encoded Ag43 protein was synthesized during agitated growth, but not in static cultures. In contrast, the flagellar protein (FliC-H21) was present in samples from static cultures, but it was undetectable by 2D-PAGE in agitated culture samples. This opposite regulatory effect on the expression of FliC and Ag43 was previously reported [63]. Interestingly, in the absence of LAA, FliC-H21 was detected in similar quantities in extracts obtained from agitated and static cultures, suggesting the presence of a regulatory crosstalk between LAA and the flagellar genes that deserves future analysis. As expected, the OMP profiles of the ΔLAA mutant did not show spots corresponding to the NmpC and Ag43 proteins (both encoded in LAA). A single spot corresponded to the Tia and LAA-encoded Hes proteins. Both proteins belong to the Heat resistant agglutinin family (Hra), and share 65% identity in their amino acid sequence [10], with nearly identical isoelectric points and molecular masses. The E045-00 strain contains two copies of the *tia* gene, localized in the LIC and LAC PAIs (Table S1). In the wt and ΔLAA strains, Tia was more abundant in the static culture samples; it remains to be determined if both *tia* genes are up-regulated under static growth. The OmpW protein which has been associated with protection against phagocytosis [64], was more abundant in the agitated culture. Based on these results, the subsequent functional experiments were performed by growing bacteria in agitated LB.

Next, we evaluated whether the deletion of LAA affected the adhesion and biofilm formation of E045-00. *In vitro* assays indicated that E045-00 and its isogenic mutant ΔLAA have similar levels of adhesion to Caco-2 and HT-29 cells after 30 min or 1 h of incubation. Furthermore, the biofilm formation of E045-00 at 24, 48 and 72 h were low and unaffected by the deletion of LAA (not shown).

Then the role of LAA in intestinal colonization was evaluated in a streptomycin-treated mouse infection model. Colonization levels of E045-00 and the ΔLAA mutant were significantly different at 2, 3, 6, 8, and 9 days post-infection, suggesting the involvement of LAA in this phenotype; however, no significant difference were observed at days 10–12 (Figure 4(b)). It is worth mentioning that E045-00 harbors the LAC PAI, where the *vpe* and *deoK* operons, which promote colonization of the murine intestine [55,56], are located. At this point, we can't rule out the possibility that genes contained within LAC or other PAIs may mask the lack of LAA.

To further evaluate the functionality of LAA in a different genetic background, we used the STEC O91:H21 V07-4-4 strain, from which a ΔLAA mutant was generated. As observed in E045-00, the deletion of LAA did not affect the adhesion to human epithelial cells and biofilm formation of V07-4-4 (not shown). The V07-4-4 produces Stx2d, the Stx subtype with the highest toxicity in the murine model [65]. Therefore, groups of streptomycin-treated mice were infected with the V07-4-4 or its ΔLAA mutant and survival of the mice was recorded. Mice infected with either V07-4-4 or its ΔLAA mutant showed signs of disease (lethargy and reduced food and water consumption) from day 2–8 after challenge. Two out of 10 (20%) and four out of 10 (40%) mice challenged with V07-4-4 or the ΔLAA mutant, respectively, survived until the end of the experiment (day 13). Nevertheless, a non-significant difference between the experimental groups was observed (*p* = 0.1621) in the survival curves (Figure 4(c)).

### Phylogenetic and population structure analyses and molecular typing

**Figure 5.**
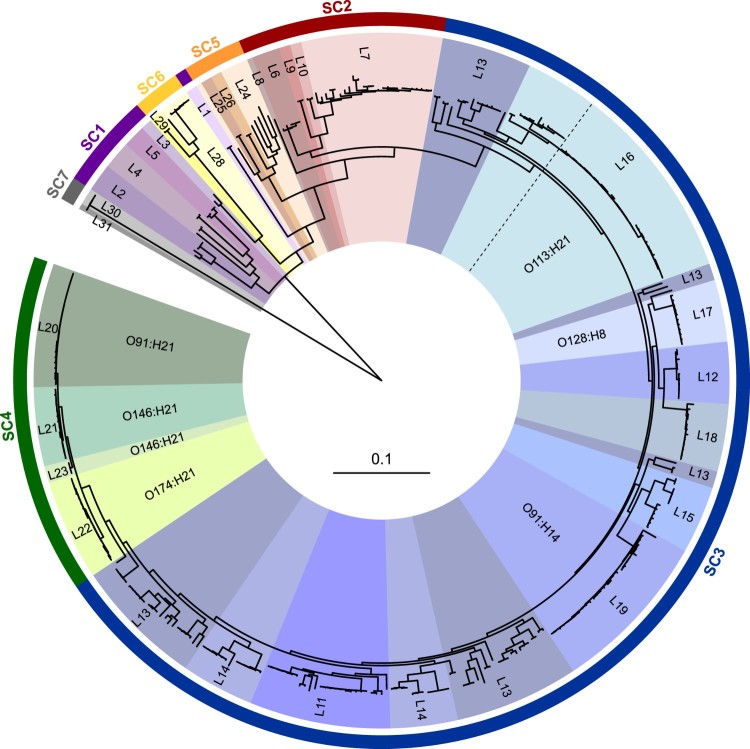
Phylogenetic relationships and population structure of LEE-negative STEC strains analyzed in this study. The maximum parsimony phylogenetic tree (midpoint rooted) is based on 3,956 core SNPs identified in 367 complete or draft genomic sequences of LEE-negative STEC and *E. coli* K-12 MG1655. The tree was built using kSNP3.1 [39] and further processed with the Interactive Tree of Life tool [77]. Sequence clusters (SC; SC1 to SC7) are indicated in the outer colored ring, which are further divided into 31 lineages (inner ring). Clinically important serotypes are shown. In general, lineages contain strains belonging to a specific serotype. In the lineage 16, branches corresponding to O113:H21 serotype are delineate with a dotted line.

We investigated the evolutionary history of the strains through phylogenetic analysis based on single nucleotide polymorphisms (SNPs) found in every genome (core SNPs). The maximum parsimony tree obtained is shown in Figure 5. Next, the population structure was assessed by a Bayesian clustering method (see methods), which identified seven sequence clusters (SCs; primary clusters) that were further subdivided into 31 lineages (L1 to L31; BAPS level 2). Most of the lineages were monophyletic and included strains belonging to a specific serotype with some exceptions, as lineage 13, which included several serotypes and ancestors localized in different branches across the tree. This analysis also allowed the identification of several sub-lineages (BAPS level 3; see below). Phylogroups and Sequence Types (STs) were also determined. A total of one hundred and six different STs were identified, all of which have been previously described (Table S1). In general, the topology of the tree was consistent with the distribution of phylogroups, serotypes and STs, but not serogroups, which in some cases (e.g. O91, O113, O174) were separated into distant branches (Figures 5 and 6). A previous study has also shown the polyphyletic nature of STEC serogroups [10].

It has been reported that among LEE-positive STEC strains from different lineages may differ in their virulence potential [20,66,67]. Moreover, it has been suggested that hypervirulent lineages containing different arrays of virulence genes correlate with the incidence and severity of diseases associated with STEC [68,69]. Furthermore, the cumulative acquisition of PAIs in LEE-positive STEC strains has been correlated with an increased ability to cause severe disease in humans, suggesting an additive or synergistic effect [17]. Therefore, we hypothesize that genome plasticity may also play an important role in the appearance of LEE-negative STEC lineages with an increased virulence potential. Thus, we attempted to determine whether there are lineages with greater virulence potential among LEE-negative STEC, and, if so, their geographical distribution. With this aim, we mapped the distribution of virulence determinants and epidemiological data onto the maximum parsimony tree. For better visualization and understanding, we focused this analysis on clinically relevant serotypes (O91:H14, O91:H21, O113:H21, O128:H8, O146:H21 and O174:H21) (Figure 6). Notably, we found concordance between the phylogeny and the virulence repertories, and, in some cases, with the geographical distribution. Figure 6.Phylogenetic relationships between LEE-negative STEC strains belonging to serotypes associated with human disease. Clusters corresponding to serotypes O48:H21, O116:H21, O113:H21 (a), O128:H8 (b), O91:H14 (c), O174:H21, O146:H21 and O91:H21 (d) were extracted from the maximum parsimony tree constructed with 3,956 core SNPs shown in Figure 5. Lineages and sub-lineages were determined using RhierBAPs [41]. Sub-lineages are indicated by colors in the branches of the tree. Distribution of virulence determinants are shown in the right panel. The presence or absence of each Mobile Genetic Element (MGE) or gene is indicated by orange or white squares, respectively. Epidemiologic data of each strain, including country, year and source of isolation is shown (see legend).

**Figure:**
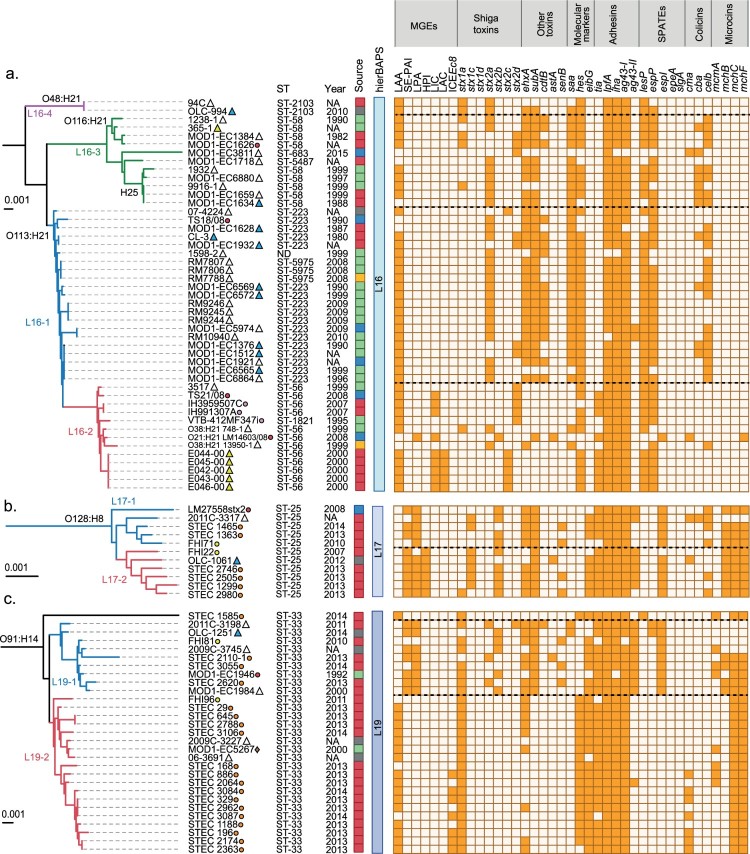

**Figure:**
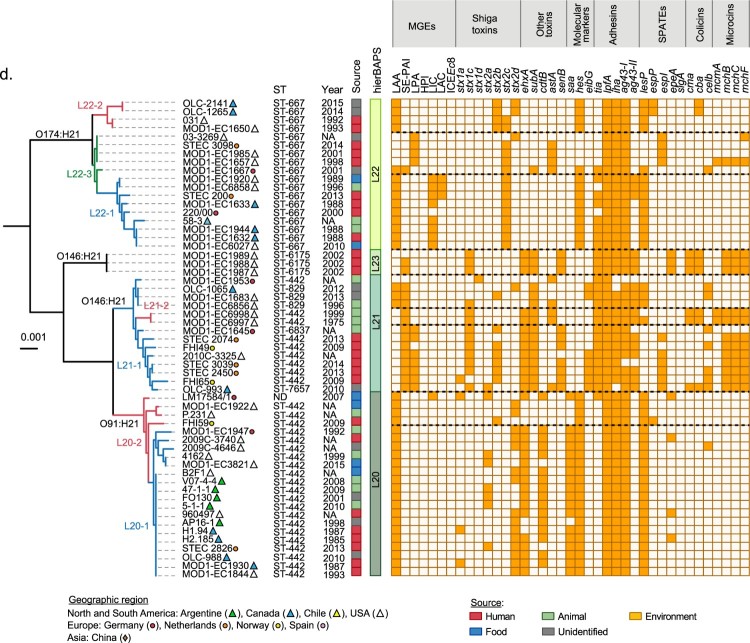

For example, O113:H21 serotype (lineage 16) was separated into two sub-lineages (Figure 6(a)); sub-lineage 16–1 included twenty-one strains from North America, nineteen O113:H21 and two non-typeable strains. In addition, sub-lineage 16–2 include strains from North and South America (8/13) and Europe (5/13). Sub-lineage 16–2 also included two O38:H21 strains and one O21:H21 strain, suggesting that these strains emerged from an O113:H21 sub-lineage 16–2 ancestor (Table S1). Most of the O113:H21 strains harbor LAA. Several strains from sub-lineage 16–2 also harbor LIC (9/13) and LAC (5/13). Regarding toxin gene profiles, strains from sub-lineage 16–1 were positive for *stx_2a_* (17/21), *stx_2d_* (4/21), *ehxA* (19/21) and *subAB* (20/21), while those from sub-lineage 16–2 were positive for *stx_2c_* (5/13), *stx_2d_* (5/13), two strains for stx*_2a_* one of which harbors an additional stx*_1a_* and one strain harboring stx*_2b_*.

The lineage 17 was associated with eleven strains, all belonging to the O128:H8 serotype, and separated into two sub-lineages (Figure 6(b)). In both sub-lineages, 17-1(5/11) and 17–2 (6/11), the majority of strains were from Europe with one North American strain present in each group. Strains from sub-lineage 17–1 harbor SE-PAI (3/5) and LPA (5/5); most of the strains from sub-lineage 17–2 harbor these PAIs, but they also include HPI (6/6). With the exception of one strain (in 10 of 11) the *mhc* cluster was present. Other virulence genes were differentially distributed, *stx_1c_* was present in all 17–2 strains and in two 17–1 sub-lineages. Moreover, *stx_2b_* was associated with 5/6 strains in 17–2 and 4/5 strains in 17-1(Table S1). Regarding *ehxA* and *subA* both were present in almost all strains of the lineage 17, with only one strain not harboring *subA* and two strains not harboring *ehxA*.

In 2013 and 2014, O91:H14 was the most prevalent STEC serotype in a prospective multicenter study conducted in the Netherlands [11]. Most of the O91:H14 strains here analyzed were isolated as part of that study. Twenty-nine strains of this serotype were clustered into lineage 19, which was divided in three sub-lineages (Figure 6(c)). Sub-lineage 19–1 grouped nine strains from North America and Europe while sub-lineage 19–2 contained nineteen strains predominantly from the Netherlands (15/19). Sub-lineage 19–3 includes a single strain. SE-PAI (7/9) and LPA (8/9) were present only in sub-lineage 19–1 and absent in 19–2 and 19-3. However, in sub-lineage 19-2, LAA and ICE*Ec*8 were observed in 16/19 and 7/19 strains, respectively. In addition, the dominant toxin genotypes of the sub-lineage 19–1 were *stx_1a_* (7/9), *stx_2b_* (5/9) and *stx_2a_* (3/9) and stx*_2d_* (1/9) were less detected. Meanwhile, stx*_1a_* was present in all strains associated with sub-lineages 19-2-and 19-3. Other virulence genes like *ehxA* and *subAB* were detected in 7/9 and 9/9 strains, respectively, in sub-lineage 19-1, while *ehxA* was less prevalent in 19–2 (9/19) and absent in 19-3; the *subAB gene* was absent in both 19–2 and 19–3 sub-lineages. The *mch* cluster was detected in all strains associated with 19–2 and 19–3 (20/20) and in 4/9 in the 19–1 sub-lineage. These results showed that the mutual exclusivity between LAA and SE-PAI/LPA may occur regardless of the serotype with the exception of five strains, one serogroup O2, three O146:H21 and one O174:H21, belonging to the linages L8, L21 and L22, respectively.

Similarly, the O174:H21 serotype (eighteen strains belonging to Lineage 22) was divided into three sub-lineages (Figure 6(d)). Sub-lineage 22–1 (9/18) grouped strains from North and South America and Europe while strains included in sub-lineages 22–2 (4/18) and 22–3 (5/18) were mainly from North America. Strains from sub-lineage 22–1 harbored LAA (9/9), LIC (8/9), LAC (3/9) and *stx_2c_* (9/9); those from sub-lineage 22–2 harbored LAA (4/4), *stx_2b_* (4/4)*_,_ stx_2c_* (2/4)*_,_ and stx_2d_* (2/4); finally, sub-lineage 22–3 harbored LPA (4/5), LAA and SE-PAI (1/5, both PAIs in the same strain), *stx_2c_* (4/5), *stx_2d_* (1/5) and *astA* (4/5).

The O146:H21 serotype was clustered into two lineages (L23 with three strains and L21 with fourteen strains; Figure 6(d)). Lineage 23 grouped strains from the USA. Lineage 21 was divided into two sub-lineages, L21-1, which grouped most of the strains (12/14), from North America and Europe and L21-2 from the USA (2/14). The genes *stx_1c,_ ehxA*, *subA* and *senB* were detected in all lineage 23 strains; SE-PAI (2/3) was detected in lineage 23; SE-PAI, LPA, *stx_1c_*, *stx_2b_*, *ehxA*, *subA* and *senB* were heterogeneously detected in sub-lineage 21-1; and SE-PAI, stx_1c_, *ehxA*, *subAB* and *astA* were detected in both strains from sub-lineage 21-2. Interestingly, as mentioned above, sub-lineage L21-1 included 3 of the 4 strains in which LAA and SE-PAI (Figure 3(a)) were detected together. Furthermore, the *mch* cluster was frequently detected in this serotype.

The O91:H21 serotype (lineage 20) was separated into two sub-lineages (Figure 6(d)) and both of which included strains from America and Europe. In contrast to the O174:H21 and O146:H21 serotypes, all but one O91:H21 strains (LM17584/1, an LIC-positive strain from Germany) were from Germany, the presence of multiple PAIs was not observed. All O91:H21 strains harbored LAA and frequently the *stx_2d_* (15/22), *ehxA* (17/22) and *cdtB* (14/22) toxin genes.

### Genome size analysis

Bacteria show substantial variations in genome size, a feature that may be linked to their lifestyle. For instance, in some bacterial pathogens there is a tendency for a narrow host range and increased pathogenicity to be associated with a reduction in genome size (reductive genome evolution) [70]. Therefore, to gain further insights into the evolution of LEE-negative STEC virulence, we examined variation in genome sizes among these strains and other *E. coli* pathotypes (Table S10). The average genome size of LEE-negative STEC strains (5.18, range 4.715.74 Mb) was significantly (*p* < 0.0001) smaller than the average of recognized human pathogens like LEE-positive STEC strains (5.37, range 4.53–5.94 Mb), but similar to typical enteropathogenic *E. coli* (tEPEC) (5.11, range 4.51–5.52 Mb) and enteroinvasive *E. coli* (EIEC) (5.06, range 4.8–5.39 Mb) strains (Figure S3a, File S4). Furthermore, an interesting finding was that genomes from human-associated LEE-negative STEC strains were significantly (*p* = 0.0052) larger (average 5.21 Mb, range 4.75–5.74 Mb) compared to those isolated from bovines (average 5.11 Mb, range 4.85–5.42 Mb), with genomes larger than 5.2 Mb almost exclusively corresponding to human isolates (Figure S3b, File S5). These observations might reflect a higher degree of adaptability in LEE-negative STEC strains that are both capable of infecting humans and colonizing their animal reservoir.

## Discussion

Using a combination of *in silico* analyses, taking advantage of the increasing data provided by massive sequencing, and experimental assays, this study provides new information regarding the evolution of virulence of LEE-negative STEC strains.

Stx is the main virulence factor of STEC; specifically, Stx1a, Stx2a and Stx2d subtypes are associated with severe disease [5]; nevertheless, none of these subtypes was prevalent in human strains compared to animal isolates (Table 1). Therefore, identification of the Stx subtype alone does not seem sufficient to predict the virulence potential of these strains. Previous studies agree with this finding [17].

While the varying configurations of virulence genes and genome sizes among LEE-negative STEC strains make it difficult to assess the virulence potential of a given strain, we found that genes such as *hes,* one of the most prevalent in LEE-negative STEC [10], may complement existing molecular risk evaluation schemes [71].

Additionally, we report the identification of three new MGEs that are distributed in different LEE-negative STEC strains. Among these, LIC and LAC PAIs contain the *tia* gene (Figure 1), an enterotoxigenic *E. coli* virulence factor [72] that has been previously associated with the presence of SE-PAI [9]. Thus, the report of two new PAIs also containing this gene is indicative that it has spread to and within STEC strains, rendering another example of a horizontal transfer event within *E. coli* pathotypes [73]. Other genes located in these PAIs, such as those encoding CDI systems, could provide competitive advantages against other bacteria. During the review of the present manuscript, Saile et al. [74] reported the identification of several genetic elements with characteristics of PAIs in a number of LEE-negative STEC strains. Among the genetic sequences reported in that study are LIC and partially LAC.

On the other hand, the identification of ICE*Ec*8 containing *sigA* is an important finding (Figure 3), as SigA participates in infant rabbit intestinal colonization by the STEC/EAEC O104:H4 strain that caused the 2011 HUS outbreak in Europe [59]. It is noteworthy that a recent study reported the identification of the *sigA* gene in 36 STEC O117:H7 strains isolated in the United Kingdom [75], suggesting that they harbor ICE*Ec*8. Thus, it is important for future studies to address the functional characterization of this novel MGE.

This is the first large-scale study showing the accumulation and diverse distribution of GIs among LEE-negative STEC strains. Thus, together with plasmids and bacteriophages, PAIs and ICEs may play important roles in the evolution of the virulence of these pathogens. Including the results of this report, six PAIs and one ICE have been shown to be harbored by LEE-negative STEC; however, it is likely that there are more MGEs yet to be identified (Figure S4).

It is worth highlighting that LAA was the most prevalent PAI among the strains (Table 1). In addition, our results suggest that LAA contributes to the intestinal colonization in streptomycin-treated mice, but its participation in virulence could not be statistically demonstrated, although a mild attenuation of the strain could be observed (Figure 4(c)). Thus, we consider that LAA's functionality deserves a more detailed investigation. Future studies, using reduced inoculums or determining the LD50, might complement these results and provide further information on the role of LAA in virulence in this model. In addition, the association of LAA with toxin genes that cause severe disease, such as *stx_1a_*, *stx_2a_*, *stx_2d_* and *cdtB* (Figure 3(b)) could be useful in STEC surveillance.

Perhaps even more important is the epidemiologic and genomic evidence supporting the notion that there are LEE-negative STEC subpopulations that are globally distributed. Specifically, phylogenetic analyses revealed that serotypes O91:H14, O91:H21, O113:H21, O128:H8, O146:H21 and O174:H21 have sub-lineages with higher virulence potential, principally related to the Stx subtype and the number of PAIs they harbor (Figure 6).

On the other hand, we identified that there is considerable variability in the genome size of LEE-negative STEC strains, as much as 1 Mb, and a tendency for larger genomes among human strains compared to those isolated from bovines. In other words, LEE-negative STEC strains harbor the repertoires of genes that allow them to live in the bovine reservoir while large genomes could reflect the gain of genes, PAIs, ICEs, plasmids and bacteriophages by which they are able to infect humans. This hypothesis could be examined in future studies with a more significant number of fully-sequenced (closed) genomes and epidemiological data.

In conclusion, the results described here support our hypothesis as to the role of PAIs play in the emergence of LEE-negative STEC strains. Therefore, this study further adds to our knowledge on the potential pathogenic mechanisms of LEE-negative STEC and lays down a base line for their epidemiological surveillance.

## Supplementary Material

Supplemental Material
