# Supplementary material for: Cumulative acquisition of pathogenicity islands has shaped virulence potential and contributed to the emergence of LEE-negative Shiga toxin-producing Escherichia coli strains
Source: Emerg Microbes Infect. 2019 Mar 29;8(1):486–502. doi: 10.1080/22221751.2019.1595985 (PMC6455142; doi:10.1080/22221751.2019.1595985)
Supplement: Supplemental Material [file TEMI_A_1595985_SM0281.zip › Supplementary Material/Supplementary Fig 1-4/Figure S1.docx]

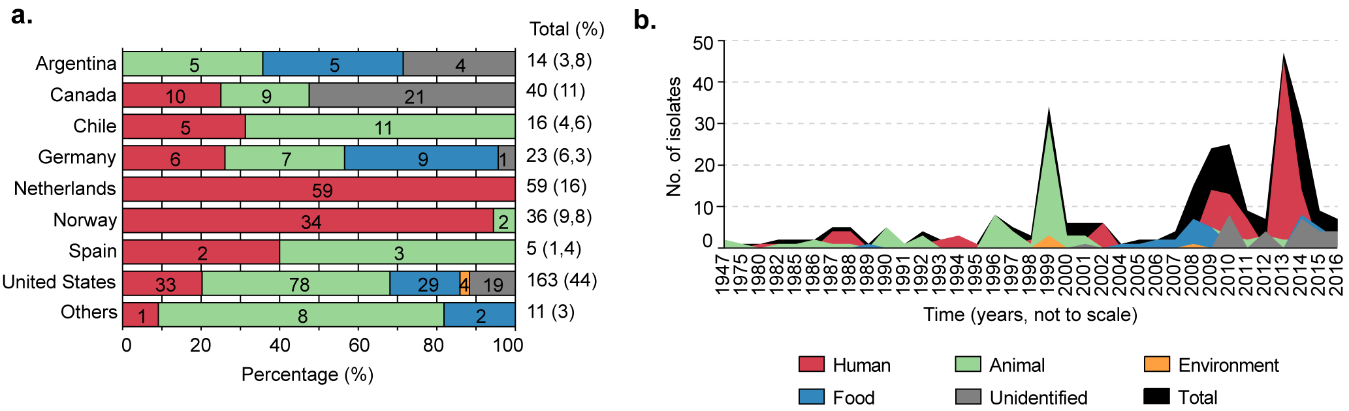


**Figure S1. Geographical and temporal distribution of LEE-negative STEC strains analyzed in this study.** The number of isolates by source (see legend) and country are indicated. **(a)** Geographical distribution. Isolates listed as from “Other” country category include: 1 clinical isolate from Belgium, 2 isolates from food from Uruguay and 8 animal isolates from Australia (1), China (1), India (1), Italy (1), Japan (2), Puerto Rico (1) and the United Kingdom (1). **(b)** Temporal distribution. Isolation date was available for 312 of the 367 analyzed strains (from 1947 to 2016). A rise in sequencing of clinical strains (human origin) is observed starting in year 2008.
