# Supplementary material for: Cumulative acquisition of pathogenicity islands has shaped virulence potential and contributed to the emergence of LEE-negative Shiga toxin-producing Escherichia coli strains
Source: Emerg Microbes Infect. 2019 Mar 29;8(1):486–502. doi: 10.1080/22221751.2019.1595985 (PMC6455142; doi:10.1080/22221751.2019.1595985)
Supplement: Supplemental Material [file TEMI_A_1595985_SM0281.zip › Supplementary Material/Supplementary Fig 1-4/Figure S2.docx]

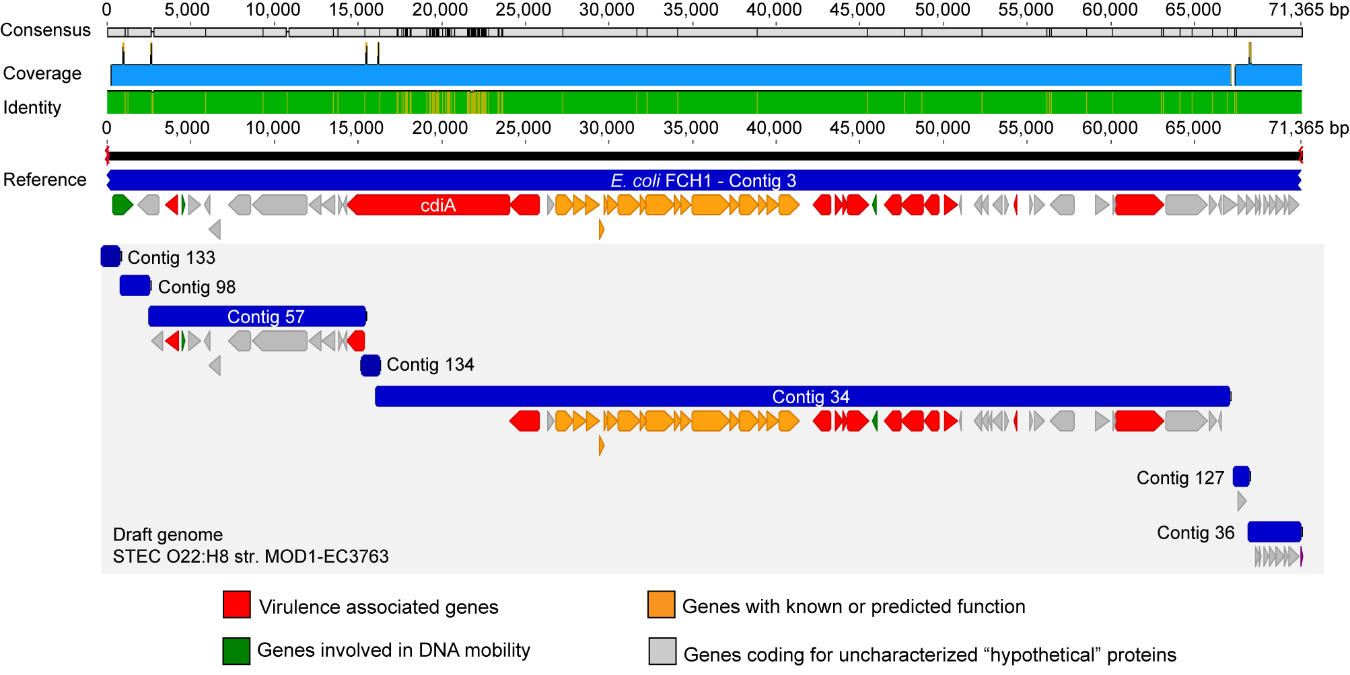


**Figure S2. Locus of Adhesion and Colonization (LAC) Pathogenicity Island identified in the draft genomes of the *E. coli* FCH1 strain (GenBank accession: AYKD01000003.1) and the STEC O22:H8 MOD1-EC3763 strain (GenBank accession: NJSE01000000).** Contigs of the WGS of the MOD1-EC3763 strain were mapped against the WGS of the FCH1 strain using Geneious software (v11.0.5; Biomatters Ltd). As the figure shown, the LAC island was found in 7 concatenated contigs (Contigs 133, 98, 57, 134, 34, 127 and 36) of the draft genome of the MOD1-EC3763 strain. Predicted genes and direction of transcription are represented as block arrows. Open reading frames (ORFs) are color coded according to gene function, as indicated by legend at the bottom. The characteristics of each ORF localized in this region are shown in Table S6.
