# Supplementary material for: Cumulative acquisition of pathogenicity islands has shaped virulence potential and contributed to the emergence of LEE-negative Shiga toxin-producing Escherichia coli strains
Source: Emerg Microbes Infect. 2019 Mar 29;8(1):486–502. doi: 10.1080/22221751.2019.1595985 (PMC6455142; doi:10.1080/22221751.2019.1595985)
Supplement: Supplemental Material [file TEMI_A_1595985_SM0281.zip › Supplementary Material/Supplementary Fig 1-4/Figure S3.docx]

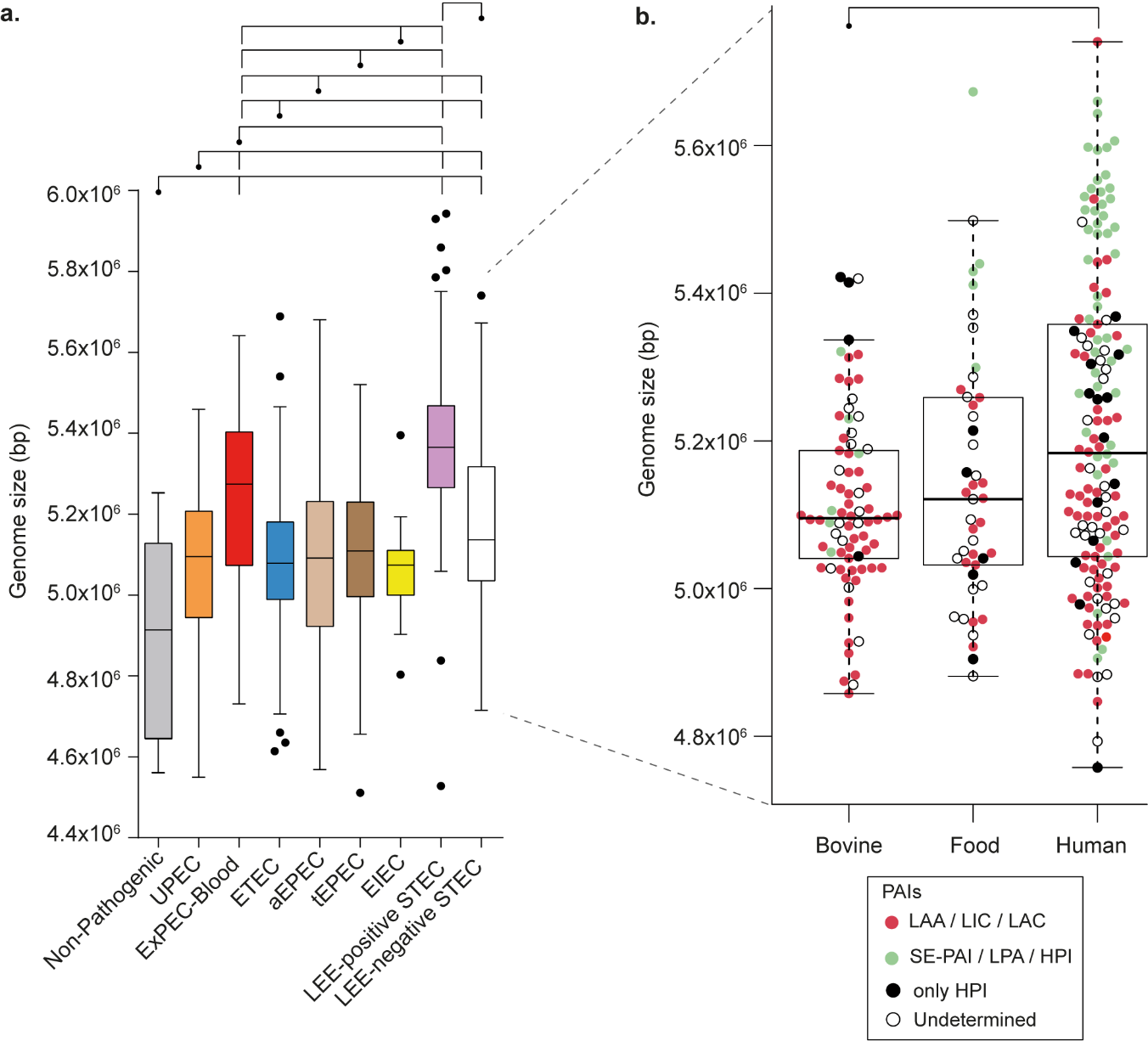


**Figure S3. Genome sizes of pathogenic and non-pathogenic *E. coli* strains. (a)** Box plots (Tukey) showing genome sizes of non-pathogenic *E. coli* strains and strains belong to six *E. coli* pathotypes; Non-Pathogenic (n=14), tEPEC (n=94), aEPEC (n=169), ETEC (n=158), EIEC (n=20), ExPEC (Blood isolates, n= 94), UPEC (n=212), LEE-positive STEC (n=188), LEE-negative STEC (n=367). Boxes indicate median and interquartile ranges (25th percentile to 75th percentile). Outliers values are shown. **(b)** Box plots (Tukey) showing genome sizes of LEE-negative STEC strains isolated from different origins. Boxes indicate median and interquartile ranges (25th percentile to 75th percentile) with whiskers extending to the 1st and 99th percentiles. Outliers values are also shown. Genomes harbored LAA and/or LAC and/or LIC are shown as red points. Genomes harbored SE-PAI and/or LPA and/or HPI are shown as green points. Genomes harbored only HPI are shown as black points. Genomes in which the above PAIs were not identified (undetermined) are shown as white points. In panels A and B, brackets indicate groups with significantly (p<0.05) larger genomes compared to those marked with a circle. Statistical differences were assessed using Kruskal-Wallis test followed by Dunn's multiple comparison test. Box plots were drawn using package beeswarm^28^ in R^29^.
