# Supplementary material for: Cumulative acquisition of pathogenicity islands has shaped virulence potential and contributed to the emergence of LEE-negative Shiga toxin-producing Escherichia coli strains
Source: Emerg Microbes Infect. 2019 Mar 29;8(1):486–502. doi: 10.1080/22221751.2019.1595985 (PMC6455142; doi:10.1080/22221751.2019.1595985)
Supplement: Supplemental Material [file TEMI_A_1595985_SM0281.zip › Supplementary Material/Supplementary Fig 1-4/Figure S4.docx]

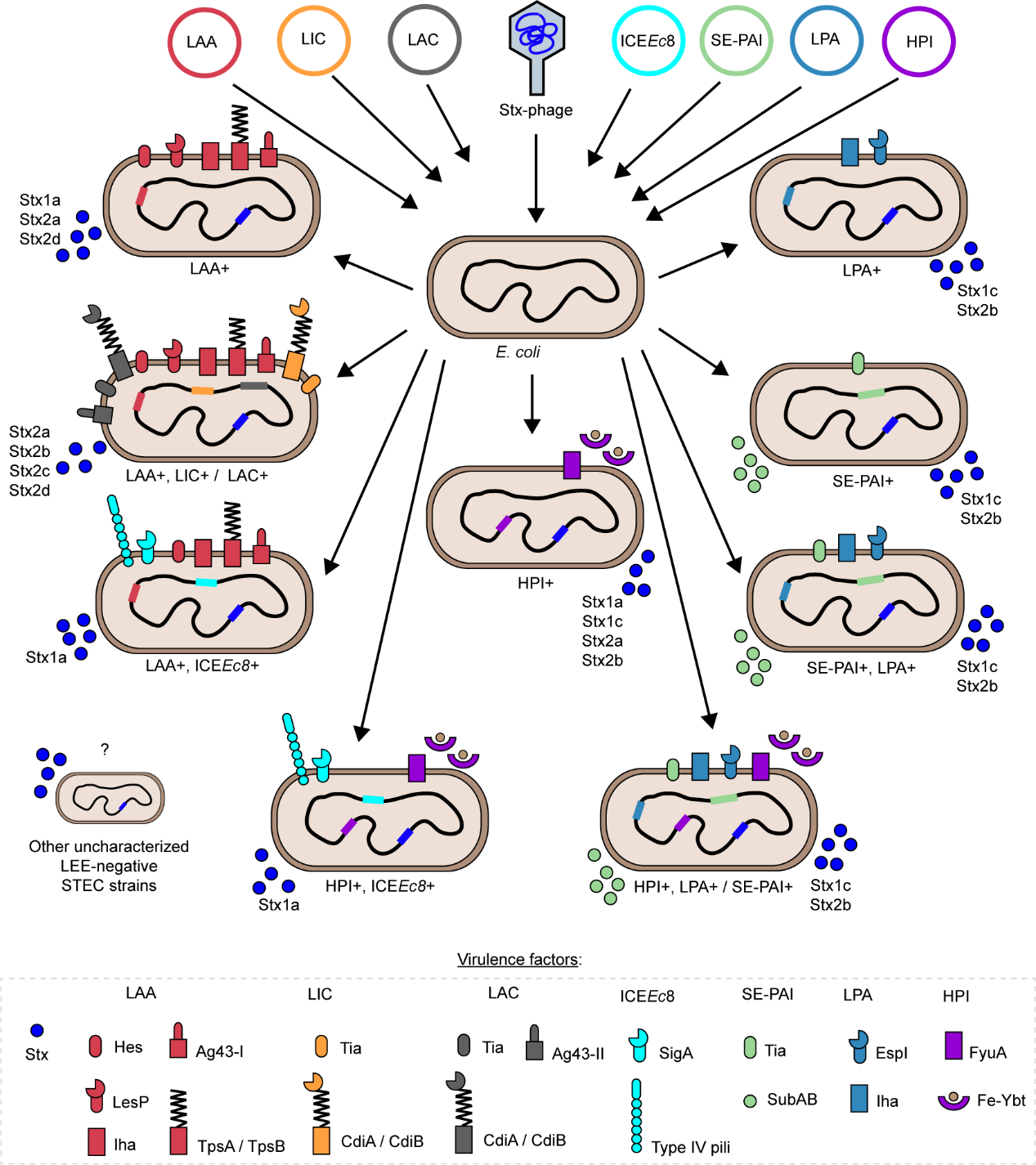


**Figure S4**. **Model for the evolution of LEE-negative STEC virulence factors, mediated by the acquisition of Stx-phages, PAIs and ICEs**. The acquisition of these mobile genetic elements (MGE) provides new molecular mechanisms to the recipient strain which increase its virulence potential and may contribute to its emergence. The figure shows the MGEs characterized so far in this subgroup of STEC. In addition, it shows how these strains may have more than one PAI in their genomes. For graphical purpose, plasmids are not shown in the figure, but it must be noted that they also have a key role in the evolution of bacteria. Virulence factors codified in each MGE are indicated in accordance with the legend at the end of the figure. The most common associated Stx profiles are also shown. LAA, Locus of Adhesion and Autoaggregation; LIC, Locus of Invasion and Contact-dependent Growth Inhibition; LAC, Locus of Adhesion and Colonization; ICE*Ec*8, Integrative and Conjugative Element of E. coli No. 8; SE-PAI, Subtilase-Encoding Pathogenicity Island; LPA, Locus of Proteolysis Activity; HPI, High-Pathogenicity Island.
