# Supplementary material for: Cumulative acquisition of pathogenicity islands has shaped virulence potential and contributed to the emergence of LEE-negative Shiga toxin-producing Escherichia coli strains
Source: Emerg Microbes Infect. 2019 Mar 29;8(1):486–502. doi: 10.1080/22221751.2019.1595985 (PMC6455142; doi:10.1080/22221751.2019.1595985)
Supplement: Supplemental Material [file TEMI_A_1595985_SM0281.zip › Supplementary Material/Supplementary Tables 1-10/Table S2.docx]

**Table S2.** Serotypes and isolation origin of LEE-negative STEC strains analyzed in this study.

| **Serogroup /**  **Serotype^1^** | **No. of strains (%)** | **Source of isolation^2^. No. of isolates (%)** | | | | |
| --- | --- | --- | --- | --- | --- | --- |
|  |  | **H** | **A** | **F** | **E** | **U** |
| O2:H25 | 4 (1,1) | 0 (0,0) | 2 (1,6) | 0 (0,0) | 0 (0,0) | 2 (4,4) |
| O6:H10 | 4 (1,1) | 1 (0,7) | 0 (0,0) | 3 (6,7) | 0 (0,0) | 0 (0,0) |
| O8:H19 | 9 (2,5) | 1 (0,7) | 3 (2,4) | 2 (4,4) | 1 (25) | 2 (4,4) |
| O15:H27 | 4 (1,1) | 1 (0,7) | 3 (2,4) | 0 (0,0) | 0 (0,0) | 0 (0,0) |
| O17/O77:H18 | 5 (1,4) | 3 (2,0) | 0 (0,0) | 1 (2,2) | 0 (0,0) | 1 (2,2) |
| O22:H8 | 10 (2,7) | 1 (0,7) | 5 (4,1) | 2 (4,4) | 0 (0,0) | 2 (4,4) |
| O76:H19 | 6 (1,6) | 4 (2,7) | 1 (0,8) | 1 (2,2) | 0 (0,0) | 0 (0,0) |
| O88:H25 | 6 (1,6) | 3 (2,0) | 3 (2,4) | 0 (0,0) | 0 (0,0) | 0 (0,0) |
| O91:H14 | 29 (7,9) | 23 (15) | 2 (1,6) | 0 (0,0) | 0 (0,0) | 4 (8,9) |
| O91:H21 | 22 (6,0) | 9 (6,0) | 6 (4,9) | 3 (6,7) | 0 (0,0) | 4 (8,9) |
| O104:H4 | 5 (1,4) | 4 (2,7) | 0 (0,0) | 0 (0,0) | 0 (0,0) | 1 (2,2) |
| O104:H7 | 6 (1,6) | 2 (1,3) | 3 (2,4) | 0 (0,0) | 0 (0,0) | 1 (2,2) |
| O104:H21 | 6 (1,6) | 3 (2,0) | 2 (1,6) | 0 (0,0) | 0 (0,0) | 1 (2,2) |
| O113:H4 | 6 (1,6) | 4 (2,7) | 0 (0,0) | 1 (2,2) | 0 (0,0) | 1 (2,2) |
| O113:H21 | 29 (7,9) | 10 (6,7) | 13 (11) | 4 (8,9) | 1 (25) | 1 (2,2) |
| O116:H21 | 4 (1,1) | 2 (1,3) | 2 (1,6) | 0 (0,0) | 0 (0,0) | 0 (0,0) |
| O117:H7 | 5 (1,4) | 5 (3,3) | 0 (0,0) | 0 (0,0) | 0 (0,0) | 0 (0,0) |
| O128ab:H2 | 6 (1,6) | 5 (3,3) | 0 (0,0) | 0 (0,0) | 0 (0,0) | 1 (2,2) |
| O128ac:H2 | 5 (1,4) | 4 (2,7) | 0 (0,0) | 1 (2,2) | 0(0,0) | 0 (0,0) |
| O130:H11 | 4 (1,1) | 1 (0,7) | 2 (1,6) | 1 (2,2) | 0 (0,0) | 0 (0,0) |
| O139:H1 | 4 (1,1) | 0 (0,0) | 2 (1,6) | 0 (0,0) | 0 (0,0) | 2 (4,4) |
| O146:H21 | 17 (4,6) | 9 (6,0) | 5 (4,1) | 0 (0,0) | 0 (0,0) | 3 (6,7) |
| O153/O178:H19 | 4 (1,1) | 0 (0,0) | 1 (0,8) | 3 (6,7) | 0 (0,0) | 0 (0,0) |
| O163:H19 | 7 (1,9) | 0 (0,0) | 5 (4,1) | 2 (4,4) | 0 (0,0) | 0 (0,0) |
| O168:H8 | 7 (1,9) | 1 (0,7) | 1 (0,8) | 5 (11) | 0 (0,0) | 0 (0,0) |
| O174:H21 | 18 (4,9) | 9 (6,0) | 3 (2,4) | 2 (4,4) | 0 (0,0) | 4 (8,9) |
| O174:H8 | 12 (3,3) | 4 (2,7) | 7 (5,7) | 0 (0,0) | 0 (0,0) | 1 (2,2) |
| O185:H7 | 4 (1,1) | 1 (0,7) | 1 (0,8) | 1 (2,2) | 0 (0,0) | 1 (2,2) |
| O187:H52 | 4 (1,1) | 2 (1,3) | 1 (0,8) | 1 (2,2) | 0 (0,0) | 0 (0,0) |
| H25 | 4 (1,1) | 1 (0,7) | 3 (2,4) | 0 (0,0) | 0 (0,0) | 0 (0,0) |
| Others ^3^ | 111 (30) | 37 (25) | 47 (38) | 12 (27) | 2 (50) | 13 (29) |
| Total | 367 | 150 (41) | 123 (34) | 45 (12) | 4 (1) | 45 (12) |

^1^ *in silico* determination using SerotypeFinder 1.1

^2^ H, Human; A, Animal; F, Food; E, Environment; U, Unidentified

^3^ Serogroups / serotypes with <4 isolates (number of strains is in parentheses): O2:H29 (1), O5:H19 (1), O6:H34 (2), O8:H9 (2), O8:H10 (1), O8:H16 (1), O8:H28 (2), O8:H49 (1), O9:H7 (1), O15:H21 (1), O21:H21 (1), O27:H30 (1), O28ac/O42:H25 (1), O36:H14 (2), O38:H21 (2), O38:H26 (1), O41:H26 (2), O45:H12 (1), O46:H38 (1), O48:H21 (2), O50/O2:H48 (1), O55:H12 (2), O71:H12 (1), O74:H42 (2), O75:H7 (2), O75:H8 (1), O75:H31 (1), O79:H7 (2), O81:H21 (1), O84:H8 (1), O88:H19 (1), O89:H9 (2), O92:H10 (1), O100:H25 (1), O100:H30 (2), O109 (2), O109:H16 (1), O110:H9 (1), O112ab:H2 (1), O112ac:H19 (1), O112ab:H21 (1), O113:H8 (1), O116:H49 (2), O117:H4 (1), O119:H4 (1), O121:H10 (1), O121:H7 (3), O130:H38 (1), O134:H38 (1), O136:H12 (1), O136:H16 (3), O138:H14 (2), O140:H21 (1), O141ac:H4 (1), O142:H38 (2), O146:H10 (1), O149:H12 (1), O151:H12 (3), O152:H19 (1), O153:H31 (1), O154:H31 (1), O166:H15 (1), O166:H28 (2), O169/O183:H46 (1), O171:H2 (2), O171:H25 (1), O174:H2 (3), O174:H25 (1), O179:H8 (1), O181:H49 (1), O183:H18 (1), Ont:H8 (1), Ont:H20 (3), OXY24:H16 (2), H8 (2), H10 (1), H11 (1), H19 (1), H21 (2).
