# Supplementary material for: Cumulative acquisition of pathogenicity islands has shaped virulence potential and contributed to the emergence of LEE-negative Shiga toxin-producing Escherichia coli strains
Source: Emerg Microbes Infect. 2019 Mar 29;8(1):486–502. doi: 10.1080/22221751.2019.1595985 (PMC6455142; doi:10.1080/22221751.2019.1595985)
Supplement: Supplemental Material [file TEMI_A_1595985_SM0281.zip › Supplementary Material/Supplementary Tables 1-10/Table S3.docx]

**Table S3.** Virulence genes identified in LEE-negative STEC strains.

| **Virulence gene** | **Function/effect** | **Reference** |
| --- | --- | --- |
| **Toxins** | | |
| *ehxA* (Plasmid-encoded O157 enterohemolysin) | Hemolysis | ^1^ |
| *subA* (Subtilase cytotoxin) | Microvascular thrombosis, endothelial damage | ^2^ |
| *astA* (Enteroaggregative heat-stable toxin 1) | Activates guanylate cyclase resulting in ion secretion | ^3^ |
| *cdtB* (Cytolethal distending toxin) | Cell cycle arrest and cytoplasmic distension | ^4^ |
| *sta1* (Heat-stable enterotoxin ST-Ia) | Induces fluid loss | ^5^ |
| *stb* (Heat-stable enterotoxin II) | Induces fluid loss | ^6^ |
| *senB* (ospD2; Plasmid-encoded enterotoxin) | Enterotoxin, unknown mechanism | ^7^ |
| **Molecular markers of LEE-negative STEC** | | |
| *saa* (STEC autoagglutinating adhesin) | Adhesion, siderophore receptor | ^8^ |
| *hes* (Hemagglutinin from STEC) | Adhesion, hemagglutination, autoaggregation, biofilm formation | ^9^ |
| *eibG* (Immunoglobulin-binding protein G) | Chain-Like adhesion phenotype | ^10^ |
| *sab* (STEC autotransporter contributing to biofilm formation) | Adhesion, biofilm formation | ^11^ |
| **Adhesin genes** | | |
| *tia* (Toxigenic invasion loci A) | Invasion and adhesion | ^12^ |
| *hra1* (Heat-resistant agglutinin 1) | Adhesion, hemagglutination, autoaggregation, biofilm formation | ^13^ |
| *lpfA* (Long polar fimbriae subunit A) | Chromosomal fimbrial adhesin | ^14^ |
| *iha* (IrgA homologue adhesin) | Adhesion | ^15^ |
| *ag43*-I and *ag43*-II (Alleles of the Antigen 43 family) | Adhesion, autoaggregation, biofilm formation | ^16^ |
| **Serine Protease Autotransporters of Enterobacteriaceae (SPATEs)** | | |
| *lesP* (LAA encoded SPATE) | Unknown function | ^9^ |
| *espP* (Extracellular serine protease plasmid pO157-encoded) | Cytotoxin, adherence | ^17^ |
| *espI* (*E. coli* secreted protease, island encoded) | Protease | ^18^ |
| *epeA* (EHEC plasmid-encoded autotransporter) | Mucinase | ^19^ |
| *pic* (Protease involved in colonization) | Mucinase, immunomodulation, colonization | ^20^ |
| *sigA* (Shigella IgA-like protease homologue) | Cytotoxin, enterotoxin | ^21^ |
| **Bacteriocins** | | |
| *cba* (Colicin B) | Colicin | ^22^ |
| *celb* (Endonuclease colicin E2) | Colicin | ^23^ |
| *cma* (Colicin M) | Colicin | ^24^ |
| *mcmA*, *mchB*, *mchC* and *mchF* (mhc cluster genes) | Chromosome-Encoded Microcins | ^25,26^ |
