# Supplementary material for: Cumulative acquisition of pathogenicity islands has shaped virulence potential and contributed to the emergence of LEE-negative Shiga toxin-producing Escherichia coli strains
Source: Emerg Microbes Infect. 2019 Mar 29;8(1):486–502. doi: 10.1080/22221751.2019.1595985 (PMC6455142; doi:10.1080/22221751.2019.1595985)
Supplement: Supplemental Material [file TEMI_A_1595985_SM0281.zip › Supplementary Material/Supplementary Tables 1-10/Table S4.docx]

**Table S4.** Shiga toxin subtypes identified among the strains analyzed in this study

| **Stx subtypes combination** | **No of strains (%)** |
| --- | --- |
| Stx1a, Stx2a | 32 (8,7) |
| Stx1c, stx2b | 30 (8,2) |
| Stx1a, stx2d | 13 (3,6) |
| Stx1a, stx2b | 8 (2,7) |
| Stx2a, Stx2a | 6 (1,6) |
| Stx2a, Stx2d | 2 (0,55) |
| Stx2b, Stx2c | 2 (0,55) |
| Stx2b, Stx2d | 2 (0,55) |
| Stx1c, Stx2a | 1(0,27) |
| Stx1c, Stx2c | 1(0,27) |
| Stx1c, Stx2d | 1 (0,27) |
| Stx2a, Stx2b | 1 (0,27) |
| Stx1a, Stx2a, Stx2d | 1(0,27) |
| Stx1c, Stx2b, Stx2b | 1 (0,27) |
| Total | 101 /367 (27,5) |
