# Supplementary material for: Cumulative acquisition of pathogenicity islands has shaped virulence potential and contributed to the emergence of LEE-negative Shiga toxin-producing Escherichia coli strains
Source: Emerg Microbes Infect. 2019 Mar 29;8(1):486–502. doi: 10.1080/22221751.2019.1595985 (PMC6455142; doi:10.1080/22221751.2019.1595985)
Supplement: Supplemental Material [file TEMI_A_1595985_SM0281.zip › Supplementary Material/Supplementary Tables 1-10/Table S5.docx]

**Table S5.** Open reading frames localized within the Locus of Invasion and Contact-dependent Growth Inhibition (LIC) of STEC O174:H21 strain MOD1-EC1633 (Accession number: NJVC00000000) identified by significant similarity (BLASTP search)

| **ORF#** | **Position (bp) *** | **Denomination in LII PAI** | **Closest informative protein match** | **No. of identical residues / Total No. of residues**  **(% Identity)** | **Accession No. of homologue** | **Function to closest related protein. Comments** |
| --- | --- | --- | --- | --- | --- | --- |
| tRNA | 1-91 | *selC*-tRNA | | | | |
|  | 72 - 95  48318- 48341 | DR1: direct repeat | TTCGACTCCTGTGATCTTCCGCCA  TTCGACTCCTGTGATCT-CCGCCA | | | |
| 1 | 395 - 1579 | Integrase | Integrase | 394/394 (100%) | WP_001218900.1 | Site-specific recombinase, phage integrase family |
| 2 | 11991 – 3034 | *sisA* | Virulence protein ShiA | 343/347 (99%) | AHA68728.1 | Attenuation of host inflammatory response |
| 3 | c3385 - 4887 | Putative membrane-associated, metal-dependent hydrolase | Putative membrane-associated, metal-dependent hydrolase | 500/500 (100%) | YP_002410067 | Unknown |
| 4 | C5062 - 5808 | *tia* | Tia invasion determinant | 248/248 (100%) | YP_002410068 | Adhesion and invasion |
| 5 | 6065 - 6216 | Integrase, fragment | putative Prophage integrase (fragment) | 52/53 (98%) | YP_002410069.1 | Gene remnant, prophage functions |
| 6 | 6410 - 6763 | hypothetical protein | hypothetical protein | 116/117 (99%) | EFJ81903.1 | Unknown |
| 7 | 7405 - 8361 | hypothetical protein | hypothetical protein | 318/318 (100%) | EHN85659.1 | Unknown |
| 8 | c8342-9271 | DNA cytosine methyltransferase | DNA cytosine methyltransferase | 309/309 (100%) | WP_001328257.1 | Unknown |
| 9 | 9845-10099 | ISRaq1-like | ISRaq1 | 74/82 (90%) | AY528232 | Transposase |
| 10 | 10312-10587 | IS629 | IS629 | 87/90 (97%) | X51586 | Transposase |
| 11 | 10587 - 11066 | IS1203E | IS1203E | 132/137 (96%) | X97542 | Transposase |
| 12 | 11658 - 13181 | hypothetical protein | Group II intron reverse transcriptase/maturase | 502/502 (100%) | YP_002406484.1 | Unknown |
| 13 | 13376 - 13750 | IS911 | IS911 | 122/125 (98%) | X17613 | Transposase |
| 14 | 14217 - 14678 | Filamentous hemagglutinin family outer membrane domain protein | Filamentous hemagglutinin family outer membrane domain protein | 153/153 (100%) | EKI34454.1 | Contact-dependent Growth Inhibition |
| 15 | 14716- 14892 | pre-toxin domain with VENN motif family protein | pre-toxin domain with VENN motif family protein | 58/58 (100%) | EKI34455.1 |  |
| 16 | c14927 - 15250 | hypothetical protein | DUF2569 domain-containing protein | 106/107(99%) | WP_077779681.1 | Unknown |
| 17 | c15463 – 15714 | hemolysin domain protein | hemolysin domain protein | 83/83 (100%) | KDU01438.1 | Contact-dependent Growth Inhibition |
| 18 | c15978 - 16412 | filamentous hemagglutinin family outer membrane protein | filamentous hemagglutinin family outer membrane protein | 144/144 (100%) | KDU01311.1 |  |
| 19 | c16876 - 17361 | hypothetical protein | hypothetical protein | 161/161 (100%) | WP_032260910.1 | Unknown |
| 20 | c17358 - 27137 | *cdiA* | *cdiA* | 3182/3259 (98%) | EKI34460.1 | Contact-dependent Growth Inhibition |
| 21 | c27150 - 28916 | *cdiB* | ShlB/FhaC/HecB family hemolysin secretion/activation protein | 588/588 (100%) | WP_077166514.1 |  |
| 22 | c29610 - 30233 | DNA-binding protein | DNA-binding protein | 207/207 (100%) | KHG70693.1 | Unknown |
| 23 | c30322 - 30834 | hypothetical protein | hypothetical protein | 169/170 (99%) | WP_001680714.1 | Unknown |
| 24 | c31403 - 31975 | hypothetical protein | hypothetical protein | 190/190 (100%) | WP_032260913.1 | Unknown |
| 25 | c32221 – 32787 | hypothetical protein | conserved hypothetical protein | 187/188 (99%) | EDV68605.1 | Unknown |
| 26 | 34274 - 35626 | hypothetical protein | hypothetical protein | 450/450 (100%) | WP_032260914.1 | Unknown |
| 27 | c36691 - 37422 | transposase | transposase | 243/243 (100%) | KHG90946.1 | Transposase |
| 28 | 38347 – 38622 | hypothetical protein | hypothetical protein | 91/91 (100%) | WP_001551885.1 | Unknown |
| 29 | 39738 - 40961 | hypothetical protein | hypothetical protein | 407/407 (100%) | WP_001107214.1 | Unknown |
| 30 | 41062 - 41946 | 50S ribosome-binding GTPase family protein | 50S ribosome-binding GTPase family protein | 294/294 (100%) | WP_032260917.1 | Unknown |
| 31 | 42578 - 42829 | hypothetical protein | transcriptional regulator, partial | 83/83 (100%) | PKZ09602.1 | Unknown |
| 32 | 42977 – 43654 | hypothetical protein | hypothetical protein | 225/225 (100%) | WP_033801393.1 | Unknown |
| 33 | 43717 - 43893 | hypothetical protein | DUF905 domain-containing protein | 58/58 (100%) | WP_033801392.1 | Unknown |
| 34 | 43983 – 44801 | hypothetical protein | DUF945 domain-containing protein | 272/272 (100%) | WP_033802047.1 | Unknown |
| 35 | 45067 - 45546 | kclA | antirestriction protein | 159/159 (100%) | WP_000706975.1 | Unknown |
| 36 | 45561 - 46037 | yeeS | DNA repair protein RadC | 158/158 (100%) | WP_001318130.1 | Unknown |
| 37 | 46124 – 46345 | yeeT | DUF987 domain-containing protein | 72/73 (99%) | WP_000692350.1 | Unknown |
| 38 | 46545 – 46724 | yeeU | antitoxin, partial | 44/45 (98%) | WP_071852595.1 | Antitoxin |
| 39 | 46834 – 47256 | hypothetical protein | DUF957 domain-containing protein | 140/140 (100%) | WP_001521668.1 | Unknown |
| 40 | 47341 - 48183 | hypothetical protein | DUF4942 domain-containing protein | 280/280 (100%) | WP_001521669.1 | Unknown |

* c: indicates ORFs transcribed on the complementary strand. Positions according to Supplementary File 1.
