# Supplementary material for: Cumulative acquisition of pathogenicity islands has shaped virulence potential and contributed to the emergence of LEE-negative Shiga toxin-producing Escherichia coli strains
Source: Emerg Microbes Infect. 2019 Mar 29;8(1):486–502. doi: 10.1080/22221751.2019.1595985 (PMC6455142; doi:10.1080/22221751.2019.1595985)
Supplement: Supplemental Material [file TEMI_A_1595985_SM0281.zip › Supplementary Material/Supplementary Tables 1-10/Table S6.docx]

**Table S6.** Open reading frames localized within Locus of Adhesion and Colonization (LAC) of the *Escherichia coli* strain FCH1 (Accession number: AYKD01000000) identified by significant similarity (BLASTP search)

| **ORF#** | **Position (bp) *** | **Denomination in LII PAI** | **Closest informative protein match** | **No. of identical residues / Total No. of residues**  **(% Identity)** | **Accession No. of homologue** | **Function to closest related protein. Comments** |
| --- | --- | --- | --- | --- | --- | --- |
| tRNA | 1 - 76 | *pheV*-tRNA | | | | |
|  | 54 - 76  71335 - 71356 | DR1: direct repeat | TTCGATTCCGAGTCCGGGCACCA  TTCGATTCCGAGTCCGGC-ACCA | | | |
| 1 | 349 - 1536 | Integrase | Integrase | 395/395(100%) | EFK51858.1 | Site-specific recombinase, phage integrase family |
| 2 | c1801-3072 | Putative membrane-associated, metal-dependent hydrolase | Putative membrane-associated, metal-dependent hydrolase | 411/423 (97%) | WP_001560767.1 | Unknown |
| 3 | c3478 - 4224 | *tia* | Tia invasion determinant | 237/248(96%) | OAC05898.1 | Adhesion and invasion |
| 4 | 4471 - 4632 | Integrase, fragment | putative Proorf5phage integrase (fragment) | 50/53 (94%) | YP_002410069.1 | Gene remnant, prophage functions |
| 5 | 4838 - 5599 | hypothetical protein | restriction endonuclease | 253/253 (100%) | WP_074435527.1 | Predicted restriction endonuclease [Defense mechanisms] |
| 6 | c5791 – 6132 | hypothetical protein | 5'-nucleotidase | 113/113 (100%) | WP_028132771.1 | Unknown |
| 7 | c6102 – 6746 | hypothetical protein | 5'-nucleotidase | 179/180 (99%) | WP_028132771.1 | Unknown |
| 8 | c7265 – 8587 | hypothetical protein | hypothetical protein | 440/440 (100%) | WP_029488460.1 | Unknown |
| 9 | c8683 – 11964 | hypothetical protein | type III restriction protein res subunit | 1093/1093 (100%) | WP_029488459.1 | DEAD-like helicases superfamily |
| 10 | c12032 – 12775 | hypothetical protein | type I restriction-modification system, M subunit, partial | 241/245 (98%) | EKI46689.1 | Unknown |
| 11 | c12839 - 13579 | hypothetical protein | DEAD/DEAH box helicase, partial | 198/201 (99%) | ONG20068.1 | Unknown |
| 12 | 13828 – 14067 | symE-like toxin | type I toxin-antitoxin system SymE family toxin | 79/79 (100%) | WP_074435529.1 | Toxin |
| 13 | c14112 – 14339 | hypothetical protein | hypothetical protein | 75/75 (100%) | PAU22824.1 | Unknown |
| 14 | c14336 – 24049 | cdiA-like | contact-dependent Growth Inhibition (cdiA) | 2747/3094 (89%) | EKI34460.1 | Contact-dependent Growth Inhibition |
| 15 | c24062 – 25834 | cdiB | ShlB/FhaC/HecB family hemolysin secretion/activation protein | 590/590 (100%) | WP_029488455.1 |  |
| 16 | 26350 – 26739 | hypothetical protein | hypothetical protein | 129/129 (100%) | WP_000833174.1 | O-island #138. Unknows, putative enzymes of fatty acid biosynthesis system (acyl carrier synthases, and reductase), unclassified putative enzymes. |
| 17 | 26804 – 27862 | methyltransferase domain-containing protein | methyltransferase domain-containing protein | 352/352 (100%) | WP_029488454.1 |  |
| 18 | 27903 – 28625 | hypothetical protein | hypothetical protein | 240/240 (100%) | WP_000263010.1 |  |
| 19 | 28622 – 29443 | putative acyltransferase | 1-acyl-sn-glycerol-3-phosphate acyltransferase | 273/273 (100%) | WP_077696946.1 |  |
| 20 | 29418 – 29675 | putative acyl carrier protein | acyl carrier protein | 85/85 (100%) | WP_001148685.1 |  |
| 21 | 29687 – 29938 | putative acyl carrier protein | acyl carrier protein | 83/83 (100%) | WP_000132059.1 |  |
| 22 | 29943 – 30524 | putative membrane protein | DNA gyrase subunit B | 193/193 (100%) | WP_001442994.1 |  |
| 23 | 30521 – 31879 | putative AMP-binding protein | AMP-dependent synthetase | 452/452 (100%) | WP_001077064.1 |  |
| 24 | 31866 – 32219 | putative acyl carrier protein dehydratase | putative (3R)-hydroxymyristoyl-[acyl carrier protein] dehydratase | 117/117 (100%) | OSK21478.1 |  |
| 25 | 32210 – 33886 | putative glycosyl transferase | acyltransferase | 558/558 (100%) | WP_029488452.1 |  |
| 26 | 33890 – 34312 | putative thioesterase | acyl-CoA thioesterase | 119/119 (100%) | WP_029488451.1 |  |
| 27 | 34309 – 34914 | *lolA* | outer membrane lipoprotein carrier protein LolA | 201/201(100%) | WP_000670563.1 |  |
| 28 | 34952 – 37201 | Putative membrane protein | membrane protein | 745/749 (99%) | WP_000180163.1 |  |
| 29 | 37198 – 37782 | hypothetical protein | DUF3261 domain-containing protein | 194/194 (100%) | WP_000597701.1 |  |
| 30 | 37784 – 38953 | putative beta-ketoacyl synthase | beta-ketoacyl-[acyl-carrier-protein] synthase II | 389/389 (100%) | WP_001350102.1 |  |
| 31 | 38950 – 39414 | putative dehydratase | 3-hydroxy-fatty acyl-ACP dehydratase | 153/154 (99%) | WP_000020242.1 |  |
| 32 | 39414 – 40145 | *fabG* | 3-oxoacyl-ACP reductase FabG | 243/243 (100%) | WP_000091658.1 |  |
| 33 | 40142 – 41371 | putative beta-ketoacyl synthase | beta-ketoacyl-ACP synthase II | 409/409 (100%) | WP_000198472.1 |  |
| 34 | c42211 – 43242 | putative transcriptional regulatory protein VpeR | LacI family DNA-binding transcriptional regulator | 343/343 (100%) | WP_000416157.1 | vpe operon encoding an EII complex of the phosphotransferase (PTS) system. Urovirulence and Fitness *In Vivo* |
| 35 | 43513 – 43956 | putative PTS system-specific IIA component VpeA | PTS ascorbate transporter subunit IIA | 147/147(100%) | WP_000916811.1 |  |
| 36 | 44972 – 44259 | putative PTS system-specific IIB component VpeB | PTS ascorbate transporter subunit IIB | 95/95 (100% | WP_000705928.1 |  |
| 37 | 44272 – 45528 | putative PTS system-specific IIC component VpeC | PTS ascorbate transporter subunit IIC | 418/418 (100%) | WP_000345347.1 |  |
| 38 | c45744 – 45965 | transposase | transposase | 73/73 (100%) | EYZ96274.1 | transposase |
| 39 | c46450 – 47463 | deoxyribose specific mutarotase DeoM | DUF4432 domain-containing protein | 337/337(100%) | WP_000107474.1 | deoK gene cluster. Promotes pathogenic *E. coli* growth during host colonization |
| 40 | c47475 – 48791 | permease DeoP | L-fucose:H+ symporter permease | 438/438 (100%) | WP_000998349.1 |  |
| 41 | c48819 – 49739 | deoxyribokinase DeoK | ribokinase | 306/306 (100%) | WP_000350265.1 |  |
| 42 | 50042 – 50824 | putative transcriptional regulator DeoQ | DeoR family transcriptional regulator | 260/260 (100%) | WP_001315616.1 |  |
| 43 | c50925 – 51041 | acetolactate synthase small subunit | putative acetohydroxy-acid synthase I small subunit | 37/38 (97%) | EEJ46496.1 | Unknown |
| 44 | c51827 – 52261 | hypothetical protein | hypothetical protein | 144/144 (100%) | WP_096271942.1 | Unknown |
| 45 | c52249 - 52656 | hypothetical protein | hypothetical protein | 135/135 (100%) | WP_001443055.1 | Unknown |
| 46 | c52910 – 53479 | hypothetical protein | inovirus Gp2 family protein | 189/189 (100%) | WP_000221502.1 | Unknown |
| 47 | 53683 – 53880 | hypothetical protein | hypothetical protein | 65/65 (100%) | WP_000236763.1 | Unknown |
| 48 | c54225 – 54365 | hemolysin activation protein | hemolysin activation protein | 46/46 (100%) | WP_071527034.1 | Haemolysin expression modulating protein |
| 49 | 55126 – 55332 | AlpA family transcriptional regulator | AlpA family transcriptional regulator | 68/68 (100%) | WP_001398320.1 | Predicted DNA-binding transcriptional regulator AlpA |
| 50 | 55419 – 56021 | hypothetical protein | hypothetical protein | 200/200 (100%) | WP_077248524.1 | Unknown |
| 51 | c56343 – 57827 | hypothetical protein | DUF3987 domain-containing protein | 494/494 (100%) | WP_077792018.1 | Unknown |
| 52 | 59058 – 59930 | *yeeP* | 50S ribosome-binding GTPase | 290/290 (100% | WP_078207435.1 | Unknown |
| 53 | 60134 – 60292 | hypothetical protein | hypothetical protein | 52/52 (100%) | WP_001332431.1 | Unknown |
| 54 | 60303 – 63149 | *ag43*-II | autotransporter domain-containing protein | 948/948 (100%) | WP_029488448.1 | Bacterial adhesion and biofilm formation |
| 55 | 63270 – 65786 | hypothetical protein | hypothetical protein | 838/838 (100%) | WP_029488447.1 | Unknown |
| 56 | 65862 – 66317 | *aec69* | hypothetical protein | 151/151 (100%) | WP_000581504.1 | Unknown |
| 57 | c66438 – 66626 | hypothetical protein | DUF905 domain-containing protein | 63/63 (100%) | WP_042036194.1 | Unknown |
| 58 | 66729 – 67547 | hypothetical protein | DUF945 domain-containing protein | 272/272 (100%) | WP_029488446.1 | Unknown |
| 59 | 67602 – 68087 | *kclA* | antirestriction protein | 159/161 (99%) | WP_000849582.1 | Unknown |
| 60 | 68103 – 68579 | *yeeS* | putative radC-like protein YeeS | 158/158 (100%) | ABE06628.1 | Unknown |
| 61 | 66648 – 68869 | *yeeT* | DUF987 domain-containing protein | 73/73 (100%) | WP_000692329.1 | Unknown |
| 62 | 68869 – 68982 | hypothetical protein | hypothetical protein | 37/37 (100%) | WP_000488318.1 | Unknown |
| 63 | 69134 – 69400 | *yeeV* | antitoxin, partial | 88/88 (100%) | KYR89313.1 | antitoxin |
| 64 | 69490 – 69864 | *yeeU* | toxin | 124/124 (100%) | WP_000854753.1 | toxin |
| 65 | 69861 – 70349 | hypothetical protein | hypothetical protein | 162/162 (100%) | WP_000777541.1 | Unknown |
| 66 | 70361 – 70558 | hypothetical protein | DUF957 domain-containing protein | 65/65 (100%) | WP_001317562.1 | Unknown |
| 67 | 70655 - 71224 | hypothetical protein | DUF4942 domain-containing protein | 188/189 (99%) | WP_001290240.1 | Unknown |

* c: indicates ORFs transcribed on the complementary strand. Positions according to Supplementary File 2.
