# Supplementary material for: Cumulative acquisition of pathogenicity islands has shaped virulence potential and contributed to the emergence of LEE-negative Shiga toxin-producing Escherichia coli strains
Source: Emerg Microbes Infect. 2019 Mar 29;8(1):486–502. doi: 10.1080/22221751.2019.1595985 (PMC6455142; doi:10.1080/22221751.2019.1595985)
Supplement: Supplemental Material [file TEMI_A_1595985_SM0281.zip › Supplementary Material/Supplementary Tables 1-10/Table S7.docx]

**Table S7.** Open reading frames localized within the Integrative and Conjugative Element of STEC O117:H7 strain FHI72 (ICE*Ec*8; GenBank assembly accession: GCA_000939255.1) identified by significant similarity (BLASTP search)

| **ORF#** | **Position (bp) *** | **Denomination in ICE*Ec7*** | **Closest informative protein match** | **No. of identical residues / Total No. of residues**  **(% Identity)** | **Accession No. of homologue** | **Function to closest related protein. Comments** |
| --- | --- | --- | --- | --- | --- | --- |
| tRNA | 1 - 76 | *pheV*-tRNA | | | | |
|  | 26 - 77  86457 - 86508 | DR: direct repeat | AGGGGATTGAAAATCCCCGTGTCCTTGGTTCGATTCCGAGTCCGGGCACCAC | | | |
| 1 | c275 - 1231 | Integrase | Recombinase XerD | 314/318 (98%) | WP_000777206.1 | DNA replication and recombination. |
| 2 | c1301 -2911 | Helicase / relaxase | Integrating conjugative element relaxase, PFGI-1 class | 528/532 (99%) | WP_001625914.1 | Putative relaxase required for transfer by a subclass of integrating conjugative elements |
| 3 | c2904-4412 | ATP-dependent helicase | ATP-dependent helicase | 502/502 (100%) | WP_001120272.1 | Superfamily I DNA. Replication, recombination and repair |
| 4 | C4540 - 4971 | Hypothetical protein | Hypothetical protein | 117/118 (99%) | EZJ31791.1 | Unknown |
| 5 | 4952 - 5182 | Hypothetical protein | Hypothetical protein | 76/76 (100%) | WP_001138606.1 | Unknown |
| 6 | 5191 - 5385 | Hypothetical protein | Hypothetical protein | 63/64 (98%) | WP_042104503.1 | Unknown |
| 7 | 5395 - 5631 | Hypothetical protein | Hypothetical protein | 72/78 (92%) | WP_074014985.1 | Unknown |
| 8 | 5664 - 6353 | Hypothetical protein | Hypothetical protein | 73/78 (94%) | WP_021538137.1 | Unknown |
| 9 | 6354 - 6788 | PilM protein | PilM protein | 144/144 (100%) | WP_033807106.1 | Type IV pilus system |
| 10 | 6997 - 8598 | PilN protein | PilN protein | 533/533 (100%) | WP_000713048.1 |  |
| 11 | 8610 - 9917 | PilO protein | PilO protein | 431/431 (100%) | WP_001169832.1 |  |
| 12 | 9907 - 10368 | PilP protein | PilP protein | 152/153 (99%) | WP_000543768.1 |  |
| 13 | 10378 - 11898 | PilQ protein | PilQ protein | 506/506 (100%) | WP_001115303.1 |  |
| 14 | 11900 - 13000 | PilR protein | Hypothetical protein | 366/366 (100%) | WP_000876118.1 |  |
| 15 | 13052 - 13585 | PilS protein | PilS protein | 177/177 (100%) | WP_001011155.1 |  |
| 16 | 13644 - 14120 | PilT protein | Hypothetical protein | 158/158 (100%) | WP_000871802.1 |  |
| 17 | 14127 - 14783 | Hypothetical protein | Hypothetical protein | 214/216 (99%) | WP_001292581.1 |  |
| 18 | 14780 - 15997 | PilV protein | PilV protein | 404/405 (99%) | WP_016237204.1 |  |
| 19 | c15994 - 16227 | Hypothetical protein | Shufflon system plasmid conjugative transfer pilus tip adhesin PilV | 74/74 (100%) | WP_106482758.1 |  |
| 20 | 16282 - 17406 | Hypothetical protein | Shufflon-specific DNA recombinase | 374/374 (100%) | WP_001672437.1 |  |
| 21 | 17967 - 18788 | TraE protein | Hypothetical protein | 273/273 (100%) | WP_033808608.1 |  |
| 22 | 18878 - 20080 | TraF protein | F plasmid transfer operon, TraF, protein | 400/400 (100%) | WP_000979995.1 |  |
| 23 | 20442 - 24299 | SigA | Exported serine protease SigA | 1267/1285 (98%) | AAF67320.1 | Exported cytopathic protease involved in intestinal fluid accumulation |
| 24 | 24872 - 25894 | IS21 family transposase IS100 | IS21 family transposase IS100 | 340/340 (100%) | WP_000255944.1 | Transposase |
| 25 | 25894 - 26673 | Transposase | Transposase | 259/259 (100%) | WP_001323403.1 | Transposase |
| 26 | 26712 - 27089 | Hypothetical protein | Single-stranded DNA-binding protein | 114/115 (99%) | WP_001682055.1 | Unknown |
| 27 | 27136 - 28059 | Transposase | Hypothetical protein | 307/307 (100%) | WP_033808929.1 | Transposase |
| 28 | 28133 - 28501 | Transposase | Transposase | 122/122 (100%) | WP_096955009.1 | Transposase |
| 29 | 28655 - 29233 | PilL protein | PilL protein | 192/192 (100%) | WP_032308781.1 | Conjugative transfer region protein |
| 30 | 29230 - 2997 | Hypothetical protein | Hypothetical protein | 255/255 (100%) | WP_032308782.1 | Unknown |
| 31 | 30002 - 30724 | Hypothetical protein | TIGR03759 family integrating conjugative element protein | 239/240 (99%) | WP_000782640.1 | Unknown |
| 32 | 30703 - 31365 | Hypothetical protein | Transglycosylase | 219/220 (99%) | WP_032308783.1 | Unknown |
| 33 | 31371 - 31892 | Hypothetical protein | Integrating conjugative element protein | 168/168 (100%) | WP_033807331.1 | Unknown |
| 34 | 31892 - 32104 | Hypothetical protein | Restriction endonuclease | 30/30 (100%) | WP_032308785.1 | Unknown |
| 35 | 32101 - 32490 | Hypothetical protein | Restriction endonuclease | 128/129 (99%) | WP_032308785.1 | Unknown |
| 36 | 32504 - 33292 | Hypothetical protein | Hypothetical protein | 262/262 (100%) | WP_032308786.1 | Unknown |
| 37 | 33285 - 35402 | TraG protein | Conjugative coupling factor TraD, PFGI-1 class | 701/705 (99%) | WP_062864066.1 | Conjugative coupling factor |
| 38 | 35383 - 36144 | Hypothetical protein | TIGR03747 family integrating conjugative element | 249/253 (98%) | WP_000796666.1 | Unknown |
| 39 | c36401 - 36559 | Hypothetical protein | Hypothetical protein | 51/52 (98%) | WP_032308788.1 | Unknown |
| 40 | c36826 - 36948 | Hypothetical protein | Hypothetical protein | 38/39 (97%) | WP_000997863.1 | Unknown |
| 41 | 37094 - 37435 | Hypothetical protein | Hypothetical protein | 113/113 (100%) | WP_000492099.1 | Unknown |
| 42 | 37435 - 37677 | Hypothetical protein | TIGR03758 family integrating conjugative element protein | 80/80 (100%) | WP_032308789.1 | Unknown |
| 43 | 37708 - 38085 | Hypothetical protein | TIGR03745 family integrating conjugative element | 125/125(100%) | WP_001681767.1 | Unknown |
| 44 | 38105 - 38470 | Hypothetical protein | TIGR03750 family conjugal transfer protein | 120/121 (99%) | WP_001681768.1 | Unknown |
| 45 | 38467 - 39114 | Hypothetical protein | TIGR03746 family integrating conjugative element protein | 215/215 (100%) | WP_000086084.1 | Unknown |
| 46 | 39111 - 40019 | Hypothetical protein | TIGR03749 family integrating conjugative element protein | 300/302 (99%) | WP_001681769.1 | Unknown |
| 47 | 40009 - 41487 | Hypothetical protein | TIGR03752 family integrating conjugative element protein | 491/492 (99%) | WP_001681770.1 | Unknown |
| 48 | 41505 - 41918 | Hypothetical protein | TIGR03751 family conjugal transfer lipoprotein | 135/137 (99%) | WP_032308793.1 | Unknown |
| 49 | 41918 - 44734 | Type VI secretion protein | Conjugative transfer ATPase | 931/938 (99%) | WP_001681772.1 | F pilus assembly Type-IV secretion system for plasmid transfer |
| 50 | 44731 - 45117 | Hypothetical protein | Hypothetical protein | 127/128 (99%) | WP_032308795.1 | Unknown |
| 51 | c45363 - 45536 | Hypothetical protein | Hypothetical protein | 57/57 (100%) | WP_033807339.1 | Unknown |
| 52 | 45508 - 47598 | Iha protein | Bifunctional enterobactin receptor/adhesin protein | 686/696 (99%) | WP_001223350.1 | Siderophore and adhesin |
| 53 | 47825 - 48223 | Hypothetical protein | TIGR03757 family integrating conjugative element protein | 132/132 (100%) | WP_033807341.1 | Unknown |
| 54 | 48220 - 49194 | Hypothetical protein | TIGR03756 family integrating conjugative element protein | 324/324(100%) | WP_033807342.1 | Unknown |
| 55 | 49203 - 50636 | Hypothetical protein | Integrating conjugative element protein | 475/476 (99%) | WP_033807343.1 | Unknown |
| 56 | 50629 - 50994 | Hypothetical protein | Hypothetical protein | 121/121 (100%) | WP_001681777.1 | Unknown |
| 57 | 50998 - 52500 | TraG protein | Conjugal transfer protein TraG | 500/500 (100%) | WP_033807344.1 | Conjugal transfer protein |
| 58 | c52547 - 52846 | Hypothetical protein | Hypothetical protein | 97/99 (98%) | WP_077953133.1 | Unknown |
| 59 | 53383 - 53604 | Hypothetical protein | Hypothetical protein | 73/73 (100%) | WP_033807345.1 | Unknown |
| 60 | c53948 - 54292 | Hypothetical protein | Hypothetical protein | 113/114 (99%) | WP_033807353.1 | Unknown |
| 61 | 54952 - 55494 | Hypothetical protein | Hypothetical protein | 131/133 (98%) | WP_001672448.1 | Unknown |
| 62 | 55665 - 56045 | Hypothetical protein | Hypothetical protein | 126/126 (100%) | WP_001681645.1 | Unknown |
| 63 | 56146 - 56625 | Hypothetical protein | Hypothetical protein | 157/159 (99%) | WP_021544261.1 | Unknown |
| 64 | 56603 - 56740 | Hypothetical protein | Hypothetical protein | 43/45 (96%) | KGM62803.1 | Unknown |
| 65 | 56818 - 57816 | Hypothetical protein | DUF1738 domain-containing protein | 325/327 (99%) | WP_001672446.1 | Unknown |
| 66 | 57927 - 58286 | Hypothetical protein | Hypothetical protein | 119/119 (100%) | WP_000287297.1 | Unknown |
| 67 | 58354 - 58692 | Hypothetical protein | Hypothetical protein | 112/112 (100%) | WP_001247170.1 | Unknown |
| 68 | 58766 - 59203 | Hypothetical protein | Hypothetical protein | 144/144(100%) | WP_001101430.1 | Unknown |
| 69 | 59311 - 59853 | Hypothetical protein | DUF1281 domain-containing protein | 175/175 (100%) | WP_021531919.1 | Unknown |
| 70 | 59919 - 60257 | IS21 family | transposition helper protein, IS21 family | 109/112 (97%) | ABV19510.1 | Unknown |
| 71 | 60380 - 61066 | Hypothetical protein | Hypothetical protein | 228/228 (100%) | WP_000206397.1 | Unknown |
| 72 | 61198 - 61929 | Hypothetical protein | Hypothetical protein | 243/243 (100%) | WP_001121871.1 | Unknown |
| 73 | 62084 - 63034 | Hypothetical protein | Hypothetical protein | 316/316 (100%) | WP_001672445.1 | Unknown |
| 74 | 63266 - 63877 | Hypothetical protein | Hypothetical protein | 203/203(100%) | WP_021531923.1 | Unknown |
| 75 | c63867 - 64148 | Hypothetical protein | Hypothetical protein | 93/93 (100%) | WP_000856156.1 | Unknown |
| 76 | c64208 - 64600 | Hypothetical protein | Hypothetical protein | 130/130 (100%) | WP_000423941.1 | Unknown |
| 77 | 64997 - 65356 | Hypothetical protein | DUF1281 domain-containing protein | 119/119 (100%) | WP_001672443.1 | Unknown |
| 78 | c65556 - 66335 | Transposase | Transposase | 259/259 (100%) | WP_001323403.1 | Transposase |
| 79 | c66335 - 67357 | IS21 family | IS21 family transposase IS100 | 340/340 (100%) | WP_000255944.1 | Transposase |
| 80 | c67384 - 67656 | Hypothetical protein | Single-stranded DNA-binding protein, partial | 73/89 (82%) | WP_047665679.1 | Unknown |
| 81 | c67666 - 67872 | Hypothetical protein | Hypothetical protein | 68/68 (100%) | WP_032308778.1 | Unknown |
| 82 | c67966 - 68430 | Hypothetical protein | DUF3577 domain-containing protein | 154/154 (100%) | WP_001682053.1 | Unknown |
| 83 | c69055 - 71067 | DNA topoisomerase III | DNA topoisomerase III | 668/670 (99%) | WP_032308776.1 | DNA binding protein |
| 84 | c71090 - 71809 | Hypothetical protein | TIGR03761 family integrating conjugative element protein | 239/239 (100%) | WP_032308774.1 | Unknown |
| 85 | c72288 - 73574 | Hypothetical protein | helix-turn-helix domain-containing protein | 428/428 (100%) | WP_001682049.1 | Unknown |
| 86 | c73838 - 74065 | Hypothetical protein | Hypothetical protein | 75/75 (100%) | WP_021564883.1 | Unknown |
| 87 | c74062 - 74277 | Hypothetical protein | Hypothetical protein | 71/71(100%) | WP_001682047.1 | Unknown |
| 88 | c74274 - 75011 | Hypothetical protein | Hypothetical protein | 245/245 (100%) | WP_044707195.1 | Unknown |
| 89 | c75008 - 75616 | Hypothetical protein | Hypothetical protein | 202/202 (100%) | WP_021577937.1 | Unknown |
| 90 | c75603 - 75860 | Hypothetical protein | Hypothetical protein | 84/85 (99%) | WP_000063336.1 | Unknown |
| 91 | c75870 - 76118 | Hypothetical protein | Hypothetical protein | 82/82 (100%) | WP_001682043.1 | Unknown |
| 92 | c76115 - 76693 | Hypothetical protein | DUF2857 domain-containing protein | 192/192 (100%) | WP_000069531.1 | Unknown |
| 93 | c76690 - 77421 | Hypothetical protein | DUF2786 domain-containing protein | 243/243 (100%) | WP_032308772.1 | Unknown |
| 94 | c77411 - 78820 | ParB-like protein | Chromosome partitioning protein ParB | 461/469 (98%) | WP_032308770.1 | Unknown |
| 95 | c79022 - 79777 | IS408 | IS408 | 250/251 (99%) | KGM76225.1 | Insertion sequence IS408 putative ATP-binding protein |
| 96 | c79794 - 81329 | IS21 family transposase | IS21 family transposase | 511/511 (100%) | WP_021577119.1 | Transposase |
| 97 | 81331 - 81447 | Hypothetical protein | Hypothetical protein | 38/38 (100%) | CDK82228.1 | Unknown |
| 98 | c81545 - 81778 | Hypothetical protein | Chromosome partitioning protein ParB, partial | 69/73 (95%) | PNB86652.1 | Unknown |
| 99 | c81768 - 83321 | DnaB-like protein | Replicative DNA helicase | 458/462 (99%) | WP_001682040.1 | DnaB-like helicase N terminal domain |
| 100 | c83281 - 83499 | Hypothetical protein | Hypothetical protein | 66/72(92%) | WP_033806496.1 | Unknown |
| 101 | c83504 - 84352 | Hypothetical protein | Hypothetical protein | 275/300 (92%) | WP_001682039.1 | Unknown |
| 102 | 84579 - 85052 | Hypothetical protein | Hypothetical protein | 149/158 (94%) | WP_001354423.1 | Unknown |
| 103 | c85483 - 86361 | ParA family protein | ParA family protein | 291/292 (99%) | WP_001682036.1 | Chromosome partitioning protein-like protein |

* c: indicates ORFs transcribed on the complementary strand. Positions according to Supplementary File 3.
