# Supplementary material for: Cumulative acquisition of pathogenicity islands has shaped virulence potential and contributed to the emergence of LEE-negative Shiga toxin-producing Escherichia coli strains
Source: Emerg Microbes Infect. 2019 Mar 29;8(1):486–502. doi: 10.1080/22221751.2019.1595985 (PMC6455142; doi:10.1080/22221751.2019.1595985)
Supplement: Supplemental Material [file TEMI_A_1595985_SM0281.zip › Supplementary Material/Supplementary Tables 1-10/Table S8.docx]

**Table S8.** Distribution of Pathogenicity Islands and Integrative and Conjugative Elements among LEE-negative STEC serotypes.

| **PAI** | **Serotypes^1^ (No. of strains)** | **Total (%)** |
| --- | --- | --- |
|  |  |  |
| LAA | O2:H29 (1), O8:H19 (1), O8:H49 (1), O22:H8 (6), O38:H21 (1), O48:H21 (2), O74:H42 (2), O79:H7(2), O88:H25 (6), O91:H14 (17), O91:H21 (22), O104:H21 (6), O112ac:H19 (1), O113:H21 (27), O116:H21 (4), O116:H49 (1), O130:H38 (1), O134:H38 (1), O146:H10 (1), O146:H21 (3), O153/O178:H19 (4), O163:H19 (5), O166:H28 (2), O168:H8 (2), O171:H2 (4), O174:H8 (1), O174:H21 (14), O179:H8 (1), O181:H49 (1), O185:H7 (4), H10 (1), H21 (2), H25 (4) | 151 (41) |
| SE-PAI | O5:H19 (1), O22:H8 (1), O27:H30 (1), O71:H12 (1), O76:H19 (6), O81:H21 (1), O84:H8 (1), O91:H14 (7), O113:H4 (4), O128ab:H2 (6), O128ac:H2 (3), O140:H21 (1), O146:H21 (15), O151:H12 (2), O174:H8 (11), O174:H21 (1), Ont:H8 (1), H8 (2) | 65 (18) |
| LPA | O2:H29 (1), O5:H19 (1), O6:H34 (2), O15:H21 (1), O15:H27 (4), O21:H21 (1), O22:H8 (2), O38:H26 (1), O75:H8 (1), O76:H19 (1), O81:H21 (1), O91:H14 (8), O112ab:H21 (1), O113:H4 (4), O128ab:H2 (5), O128ac:H2 (5), O140:H21 (1), O146:H21 (7), O151:H12 (2), O174:H8 (6), O174:H21 (4) | 59 (16) |
| HPI | O6:H10 (4), O8:H16 (1), O15:H27 (4), O45:H12 (1), O50/O2:H48 (1), O75:H7 (2), O75:H8 (1), O76:H19 (1), O92:H10 (1), O104:H4 (5), O109 (2), O109:H16 (1), O112ac:H19 (1), O117:H7 (5), O119:H4 (1), O128ab:H2 (5), O128ac:H2 (1), O136:H16 (3), O151:H12 (3) | 42 (11) |
| LIC | O2:H9 (1), O21:H21 (1), O76:H19 (4), O91:H21 (1), O113:H4 (2), O113:H21 (8), O174:H21 (8) | 25 (7) |
| LAC | O22:H8 (1), O113:H21 (5), O174:H21 (3), O168:H8 (1), H10 (1) | 11 (3) |
| ICE*Ec*8 | O91:H14 (7), O117:H7 (5) | 12 (3) |
| ND ^2^ | O2:H25 (4), O8:H9 (2), O8:H10 (1), O8:H19 (8), O8:H28 (2), O9:H7 (1), O17/O77:H18 (5), O22:H8 (2), O28ac/O42:H25 (1), O36:H14 (2), O38:H21 (1), O41:H26 (2), O46:H38 (1), O55:H12 (2), O75:H31 (1), O88:H19 (1), O89:H9 (2), O91:H14 (3), O100:H25 (1), O100:H30 (2), O104:H7 (6), O10:H9 (1), O112ab:H2 (1), O113:H8 (1), O113:H21 (2), O116:H49 (1), O117:H4 (1), O121:H7 (3), O121:H10 (1), O130:H11 (4), O136:H12 (1), O138:H14 (2), O139:H1 (4), O141ac:H4 (1), O142:H38 (2), O146:H21 (2), O149:H12 (1), O152:H19 (1), O153:H31 (1), O154:H31 (1), O163:H19 (2), O166:H15 (1), O168:H8 (5), O169/O183:H46 (1), O171:H25 (1), O174:H25 (1), O174:H25 (1), O183:H18 (1), O187:H52 (4), H11 (1), H19 (1); Ont:H20 (3), OXY24:H16 (2) | 105 (28) |

^1^ *in silico* determination using SerotypeFinder 1.1. ^2^ ND, Not determined. Strains in which were not detected any of this PAIs and ICEs but they could have additional uncharacterized mobile elements.
