# Supplementary material for: Cumulative acquisition of pathogenicity islands has shaped virulence potential and contributed to the emergence of LEE-negative Shiga toxin-producing Escherichia coli strains
Source: Emerg Microbes Infect. 2019 Mar 29;8(1):486–502. doi: 10.1080/22221751.2019.1595985 (PMC6455142; doi:10.1080/22221751.2019.1595985)
Supplement: Supplemental Material [file TEMI_A_1595985_SM0281.zip › Supplementary Material/Supplementary Tables 1-10/Table S9.docx]

**Table S9.** Plasmids and primers used in this study

| **Plasmids** | **Description** | **Source** | | |
| --- | --- | --- | --- | --- |
| pKD4 | Template plasmid for allelic replacement | ^27^ | | |
| pKD46 | Plasmid carrying genes encoding lambda red recombinase system | ^27^ | | |
| **Primers** | **Sequence (5´to 3´) *** | **Protocol** | **Size (bp)** | **Source** |
| LAA_del_for | CGGGCACCAAATTCATATCAACGGACCT  CCACGGAGGTCCGTTTTTCGT**GTGTAGG**  **CTGGAGCTGCTTC** | Allelic replacement | 1599 | This work |
| LAA_del_rev | TTGTCAGATCCAAAAGCAAAAACCCGCC  TTGTGGGCGGGTTCTTAAGAATTAG**CAT**  **ATGAATATCCTCCTTAG** | Allelic replacement |  | This work |
| LAA_conf_for | GGTATTGCGGTATCGGTGAT | PCR | 969 | This work |
| K1 | CAGTCATAGCCGAATAGCCT | PCR |  | ^27^ |

* Priming sites 1 and 2 are indicated in bold
